# Delivering Combination Chemotherapies and Targeting Oncogenic Pathways via Polymeric Drug Delivery Systems

**DOI:** 10.3390/polym11040630

**Published:** 2019-04-05

**Authors:** Praful R. Nair

**Affiliations:** Institute for NanoBioTechnology, Johns Hopkins University, Baltimore, MD 21218, USA; pnair8@jhu.edu

**Keywords:** drug delivery, polymer, copolymer, nanoparticle, chemotherapy, paclitaxel, doxorubicin, pathway, apoptosis

## Abstract

The side-effects associated with chemotherapy necessitates better delivery of chemotherapeutics to the tumor. Nanoparticles can load higher amounts of drug and improve delivery to tumors, increasing the efficacy of treatment. Polymeric nanoparticles, in particular, have been used extensively for chemotherapeutic delivery. This review describes the efforts made to deliver combination chemotherapies and inhibit oncogenic pathways using polymeric drug delivery systems. Combinations of chemotherapeutics with other drugs or small interfering RNA (siRNA) combinations have been summarized. Special attention is given to the delivery of drug combinations that involve either paclitaxel or doxorubicin, two popular chemotherapeutics in clinic. Attempts to inhibit specific pathways for oncotherapy have also been described. These include inhibition of oncogenic pathways (including those involving HER2, EGFR, MAPK, PI3K/Akt, STAT3, and HIF-1α), augmentation of apoptosis by inhibiting anti-apoptosis proteins (Bcl-2, Bcl-xL, and survivin), and targeting dysregulated pathways such as Wnt/β-catenin and Hedgehog.

## 1. Introduction 

### 1.1. The Problem of Cancer

Accumulation of mutations in normal cells can turn them cancerous, leading to uncontrolled proliferation and insensitivity/resistance to apoptotic signals [1]. Expanding in the vicinity of normal cells and tissue architecture, they consume resources, hijack signaling of neighboring cells, and disrupt homeostasis, eventually compromising the function of the host organ. Beside proliferation, cancerous cells can turn invasive by breaching the basement membrane, migrate to the blood vessels or lymphatic systems, and colonize distant organs in a process called metastasis. Cancer is one of the leading causes of mortality worldwide [2,3,4]. There were over 18 million new cases across all cancers and nearly 10 million fatalities (both statistics omit melanoma) [3]. While there has been a steady decline in mortality rates for most cancers, a large fraction of the decline is attributable to advances in preventive strategies such as antismoking awareness and screenings [2]. 

### 1.2. Chemotherapy—Old and New

Chemotherapy is a cornerstone of current anticancer treatments and chemotherapeutic drugs usually work by targeting the higher proliferation rate of cancer cells over healthy ones. Cancer cells are more resilient than normal cells [5] and hence chemotherapeutics targeting them are particularly toxic. Chemotherapeutics consist of a large group of drugs including anti-mitotics, direct or indirect DNA damaging agents, cytotoxic antibodies, and antimetabolites [6]. In contrast to these ‘traditional’ chemotherapeutics, targeted chemotherapy consists of drugs that aim to suppress specific pathways (such as growth factor receptors and RTK inhibitors [7,8]) rather than traits such as proliferation. These drugs often target specific overexpressed or mutated proteins expressed exclusively by the cancer cells, thus improving discrimination between healthy and normal cells and reducing off-target effects. Gleevec (targeting the BCR–ABL fusion protein) [9] and vemurafenib (inhibitor of BRAF^V600E^) [10] are prominent examples of targeted chemotherapy. Surgical resection, radiation therapy, and immunotherapy are other common modes of cancer treatment.

As traditional chemotherapeutics target rapidly proliferating cells, healthy cells—such as stem cells in the bone marrow, cells lining the digestive tract, and hair follicles—can also be affected by chemotherapeutics. This leads to side effects such as anemia, fatigue, gastrointestinal distress, hair loss, infertility, and cognitive impairment [11,12,13,14,15]. These off-target effects limit the maximum dosage of chemotherapeutics that can be safely administered to the patient, termed the Maximum Tolerated Dose (MTD) [16]. Additionally, reduced numbers of bone marrow stem cells can lead to thrombocytopenia, neutropenia [17], immunosuppression, and myelosuppression [18], compromising the ability of the immune system to fight infections. Chemotherapy can also lead to toxicity in the heart [19,20], liver [21], and kidneys [22], often leading to the respective organ failures. Newer targeted chemotherapeutics, despite being more rationally designed to exploit the weaknesses of cancer cells, are not free of side-effects. Side-effects observed with traditional chemotherapy such as anemia, thrombocytopenia, neutropenia, edema, and susceptibility to infection have also been reported with targeted chemotherapy [23]. However, the magnitude of these off-targets effects are less severe than cytotoxic chemotherapy.

Determination of optimal dosage for administration of these chemotherapeutics is tremendously critical and challenging. While higher doses lead to the abovementioned side-effects, low doses are ineffective [24]. Additionally, intravenous injections of chemotherapeutics are handicapped by poor penetration of the drugs into the tumor [25]. This reduces the final dosage of chemotherapeutic delivered to the tumor which could potentially compromise their efficacy and lead to drug resistance [26]. One strategy to reduce the occurrence of chemoresistance is the coadministration of two or more chemotherapeutics. Drugs with orthogonal mechanisms of action are administered together to minimize the probability of cancer cell survival. For example, anti-mitotics such as paclitaxel have been co-administered with DNA alkylating agents such as oxaliplatin in germ cell tumors [27,28]. Combinations of 5-fluorouracil and oxaliplatin (FOLFOX) are routinely used in the treatment of numerous cancers [29,30,31]. Administration of doxorubicin, bleomycin, vinblastine, and dacarbazine (ABVD) is common for the treatment of Hodgkin’s disease [32,33]. Other notable combination chemotherapy regimens include BEACOPP [34], CAF [35], MVP [36], and MMM [37] among numerous others. However, combination chemotherapy suffers from similar toxicity and delivery issues as single drug chemotherapy [38] albeit in a decreased form [39]. 

### 1.3. Tumors Have Abnormal Vasculature

In order to sustain their continued growth, tumors need a substantial flow of nutrients and oxygen. As a consequence of this solid tumors stimulate angiogenesis, leading to higher vasculature density in tumors compared to normal tissues [38,40]. However, these blood vessels are ‘leaky’, with 200 nm to 2 µm gaps in the vessel wall [41]. This defective vessel architecture alters the permeability and leads to increased extravasation and accumulation of large molecules and particles [42], while poor lymphatic drainage from tumors leads to their retention in the tumor. This effect is called the Enhanced Permeation and Retention (EPR) effect [43] and is not exhibited by normal tissues [44]. Hence, administration of submicron-sized ‘nanoparticles’ could improve drug delivery by preferentially accumulating in the tumor [42,45,46]. Accumulation of nanoparticles in the tumor by EPR is also called ‘passive’ targeting to distinguish it from ‘active’ targeting, which is mediated by ligands on the surface of the nanoparticle. Better delivery of chemotherapeutics to the tumor would lead to fewer off-target effects and improve the MTD [47]. Nanocarriers are particularly advantageous when delivering hydrophobic drugs such as paclitaxel and cisplatin [48] that have low solubility in formulations for intravenous administration [39]. Improved drug pharmacokinetics and longer circulation time are some of the other benefits of nanocarrier-mediated delivery [49,50]. Nanoparticles that take advantage of both passive and active targeting have been a mainstay of drug delivery and is reviewed by Bazak et al. and Pearce et al. [51,52]. Some recent attempts at active targeting include targeting PD-L1 to deliver docetaxel [53], targeting CD44 in for doxorubicin delivery in invasive breast cancer [54], and using luteinizing hormone-releasing hormone peptide to target ovarian cancer cells [55]. 

The disadvantage of using submicron-sized particles is its tendency to be cleared by the immune system. Smaller-sized particles are cleared renally, and larger particles get stuck in the capillaries [56]. Cleared nanoparticles end up in the liver and spleen, contributing to off-target cytotoxicity. Addition of a polyethylene glycol (PEG) layer to the surface of nanoparticles reduces clearance by the immune system and prolongs circulation in vivo [56,57], increasing the probability of accumulation of nanoparticles in the tumor by EPR. 

## 2. Motivations behind a Polymeric Approach to Nanoparticles

Polymers and lipids are the major materials of construction for ‘organic’ nanoparticles [58]. Beside these, nanoparticles can also be assembled using silica, metals, metal oxides, and carbon nanotubes, among others. This review will focus on synthetic polymer nanoparticles.

There are several amphiphilic polymers that have been utilized to form nanoparticles (listed in Tables 1–4). The properties of these polymers can be further modified by varying the size (molecular weight), polydispersity, crystallinity, and charge. This leads to a broad range of possible attributes that can be achieved and the ability to fine-tune and tailor those properties of the nanoparticle according to the application. Polymeric nanoparticles are gaining prominence in clinic. Poly(lactic-co-glycolic acid) (PLGA) and polylactic acid (PLA) nanoparticles have received approval, while others are in various phases of clinical trials [26,59].

### 2.1. Tunability/Customizability

The biggest advantage of polymers over other materials of nanoparticle construction is their vast array of selection and the ability to vary the molecular weight of polymer chains. PEG is the most popular choice for the hydrophilic block. Polyethylethylene [60], polybutadiene [61], polydimethylsiloxane [62], polycaprolactone (PCL) [63], PLA [64], PLGA [65], and polystyrene [66] are some of the polymers that have been used as hydrophobic blocks. Beside molecular weight (chain size), other properties that can be altered include charge/zeta potential, polydispersity, and crystallinity. This enables fine-tuning the characteristics of nanoparticles such as its shape, drug-loading capacity, and its release kinetics [49,67,68,69,70,71]. In order to load more aromatic chemotherapeutics such as paclitaxel, PEG–polycaprolactone was modified to incorporate benzyl groups in the micelle core [67]. This increased the loading efficiency of paclitaxel into the cylindrical micelles. Additionally, the aromatic micelles exhibited a slower degradation rate at physiological conditions but rapidly under acidic conditions, increasing the specificity of drug release. These micelles exhibited improved cytotoxic in vitro and shrunk A549 lung tumor xenografts in vivo. Polymersomes are more stable than liposomes as they can have a thicker membrane, leading to better control over drug release [72,73]. This permits better control over the release kinetics of toxic chemotherapeutics [16]. This not only limits off-target toxicity, but also limits renal excretion of the drug. 

### 2.2. Controlled Drug Release

The hydrophobic blocks of polymers can include oxygen- (PCL and PLA [64]) and nitrogen- (polyvinylpyridine [74]) bearing groups, which can alter its degradation rate. One example is polyesters, whose degradation via hydrolysis permits controlled release of the drugs loaded in the nanoparticle [73,75]. Different polyesters have different rates of degradation, with more ‘hydrophilic’ polyesters degrading faster. Further, diblock copolymers with different degradation rates can be blended together to achieve a more precise release profile [73]. The time scale of release in vesicles can also be altered by varying the thickness of the outer shell as demonstrated by Cuomo et al. [76]. It must also be noted that the time scale for release must not be too long, as then the nanoparticles will be cleared by the immune system without releasing the payload. This will result in the exposure of the immune system to the drug and, hence, greater off-target effects. This controllable release from polymeric drug delivery system is one of its major characteristics [77,78]. 

### 2.3. Stimuli-Responsive Release

Another advantage of polymers is its customizability to be responsive to particular stimuli. Hence, polymeric nanoparticles could take advantage of the lower pH or reductive environment that exists in solid tumors, releasing their payload upon encountering these situations. This could reduce off-target effects as the release of drug under physiological conditions is minimized. Slow release of drugs by nanocarriers while in the bloodstream also reduces drug excretion via urine. While physiological pH is 7.4, altered metabolism in tumors leads to a significantly lower pH of 6.5. Nanoparticles sensitive to pH [79,80,81], redox conditions [82], reducing agents [83], hydrogen peroxide [84], and certain overexpressed enzymes (such as matrix metalloproteinase-2 [85,86]) have all been developed, leading to delivery systems that are stable in circulation but degrade upon accumulating in the tumor. Additional stimuli can include light and temperature [87,88,89]. Cuomo et al. created chitosan-coated liposomes that were stable in the mouth and stomach environments but released encapsulated curcumin in the intestinal phase [90]. Such demonstrations could have far-reaching applications in improving drug delivery via the oral route. 

Hydrogen peroxide is a byproduct of tumor metabolism [91] and is found in higher levels in the tumor than normal tissues. Vesicles responsive to hydrogen peroxide contained propylene sulfide as the hydrophobic block, whose oxidization destabilized the assembly [84]. PEG-Poly(2-vinylspyridine) polymersomes similarly destabilize in response to low pH [80]. Stimuli-responsive groups can also be inserted between the hydrophobic and hydrophilic blocks. Upon encountering the stimulus, the linkage between the two blocks is destabilized, leading to disassembly of the nanoparticle. The incorporation of disulfide (–S–S–) group between the two blocks makes them susceptible to reducing agents (including glutathione, cysteine, and dithiothreitol) [83]. An in-depth review of stimuli-responsive nanocarriers can be found in Ganta et al. [92].

## 3. Polymeric Nanoparticles

A detailed description of the polymers and preparation of polymeric nanoparticles is beyond the scope of this review. An in-depth description of polymeric nanoparticles, their formation, properties, and some applications can be found in other reviews [73,75,93,94,95,96,97,98,99]. Instead, this section will focus on providing a very brief introduction to the different polymers and the methods of assembling them into nanoparticles.

Both, natural (such as albumin and heparin) and synthetic polymers, including PEG, PCL, PLGA, PLA, polybutadiene, and polyethylethylene, among many others, have been utilized for synthesizing nanoparticles. Another mode of classification is by whether the drug to be encapsulated in the nanoparticle is conjugated to the polymer or not. Abraxane, an albumin-paclitaxel conjugate has been successful in clinic for the treatment of breast and non-small cell lung cancer, among others [100,101]. PEG is the most common choice for the hydrophilic block as it helps nanoparticles circulate longer. The presence of a PEG layer on the surface reduces opsonization of antibodies [59,102]. This reduces clearance of nanoparticles by the immune system, increasing circulation time and accumulation in the tumor [26]. Besides synthetic polymers, biocompatible polymers, such as the polysaccharides, chitosan, and alginate, have also been used extensively to form nanoparticles and are reviewed in [103,104,105,106]. Their low immunogenicity and biodegradability have led to the use of chitosan-based nanoparticles in drug delivery [103]. 

Self-assembly of amphiphilic diblock copolymers to form polymeric nanoparticles can be performed in a number of ways: solvent evaporation [67,75], thin-film rehydration [107], solvent injection, and dialysis [107]. In addition to the above, temperature and shear applied (extrusion) during formation also influences final shape of assembly. Other methods of formation of polymeric nanoparticles include salting-out, nanoprecipitation [99], and electro-spraying to form core–shell nanoparticles [108].

The shape of an assembly is a key determinant of properties such as drug loading capacity and circulation time in vivo [75]. Passive diffusion can be used to load hydrophobic drugs and dyes, while hydrophilic drugs can be loaded using a pH gradient method [109]. 

Beside chemotherapeutics, nanoparticles can also be loaded with contrast agents for imaging and diagnostic applications [110] or antisense oligonucleotides [111,112]. This review will focus almost exclusively on the applications of synthetic polymer nanoparticles in the context of cancer therapies at the preclinical stage. Two applications of interest will be covered in this review:

**a.** Delivering combination (two or more therapeutic agents: drugs or siRNAs) therapies to different types of cancer in Section 4. 

**b.** Targeting specific pathways to reduce tumor growth or increase cell death in Section 5.

## 4. Delivering Combination Therapies

**Figure 1:**
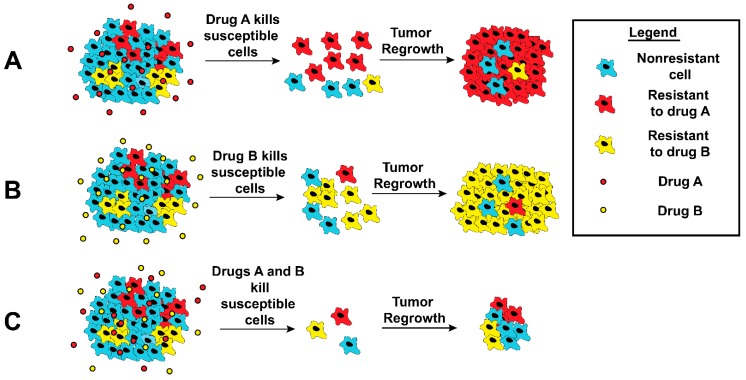
Schematic illustration of response by tumor cells following chemotherapy. Tumors are heterogeneous and consist of many subpopulations (here blue, red, and green) A and B. When cells are treated with a chemotherapeutic (**A** or **B**), the respective resistant subpopulations proliferate and eventually dominate. (**C**) Treatment with multiple drugs could eliminate this selectiveness and reduce the occurrence of resistance.

While single drugs such as Paclitaxel and Doxorubicin have enjoyed great success in clinic, development of resistance to these drugs are common. In order to suppress the emergence of resistant cells, administration of combination chemotherapy has been a commonplace for many cancers [113,114]. The underlying principle is to use cytotoxic drugs that utilize orthogonal modes of action, reducing the probability of emergence of a resistant cancer cell [115,116]. When a tumor consisting of many subpopulations is treated with a single drug, it promotes the selection and emergence of resistant cells at the expense of other tumor cells (Figure 1A,B). Treatment with two (or more) drugs ensures that no subpopulation has a selective advantage (Figure 1C). Additionally, synergy between the two drugs may also occur, where the simultaneous administration of the two drugs provides greater benefit than the individual administration of drugs combined. Such synergy between two drugs can be the result of complimentary or facilitating effects [116]. The benefits of using combination therapy over single-drug therapy are, hence, well established in clinic. 

### 4.1. Paclitaxel Combinations

Paclitaxel (abbreviated as TAX) is a routinely administered hydrophobic chemotherapeutic [117,118] and is one of the most successful chemotherapeutics [101]. It prevents cell division at the metaphase–anaphase transition by stabilizing microtubules, causing cells to undergo growth arrest and apoptosis [119,120]. Combinations of paclitaxel with other chemotherapeutics have proved an attractive and feasible solution [116]. A summary of the paclitaxel-based combinations described in this subsection is given in Table 1.

#### 4.1.1. Paclitaxel–Doxorubicin Combinations 

Being two of the most popular chemotherapeutics, combinations of paclitaxel and doxorubicin have been a focal point of drug delivery investigations [121,122,123,124]. Lv et al. demonstrated delivery by nanoparticles self-assembled from triblock copolymers [125]. Nanoparticles from PEG–poly(l-glutamic acid)-b-poly(l-lysine) were loaded with paclitaxel and doxorubicin. The triblock polymer architecture meant three different ‘zones’ in the nanoparticle, each with a purpose. Presence of a PEG layer on the outside prolonged circulation, loading of doxorubicin was made possible due to the electrostatic interaction provided poly glutamic acid (middle zone), and a hydrophobic core in the innermost part loaded paclitaxel. These nanoparticles exhibited selective release at low pH. These nanoparticles demonstrated efficacy against A549 lung cancer cells in vitro and in A549 tumor xenograft in vivo. Biodistribution at different time points showed doxorubicin fluorescence in the tumor at 10 h and 24 h postinjection, while strong hepatic signal was observed at 3 h postinjection. Immunohistochemistry of treated tumor sections revealed increased levels of DNA damage and apoptosis in tumor cells, explaining the observed in vivo efficacy. Wang et al. used PEG–PLGA nanoparticles to deliver paclitaxel and doxorubicin to three different cell lines: A549 lung, B16 mouse melanoma, and HepG2 hepatocellular carcinoma (HCC) cells [126]. The combination nanoparticles showed high uptake and produced the most cytotoxicity compared to single drug controls, with a doxorubicin: paclitaxel ratio of 2:1 being the optimal one. 

Docetaxel, a microtubule stabilizer similar to paclitaxel, has also been subject to similar attempts. Delivery of docetaxel and doxorubicin was demonstrated using PEG–PCL micelles [127]. PEG–PCL diblock copolymers conjugated to wither docetaxel or doxorubicin via disulfide bonds were mixed in a 1:1 ratio and self-assembled in water to yield micelles. In vitro efficacy was demonstrated in MCF-7 breast cancer cells along with low hemolytic (erythrocyte rupturing) activity. Zhang et al. loaded PEG–PLGA nanoparticles with docetaxel and doxorubicin [128]. To facilitate targeting to prostate cancer cells, these nanoparticles were surface-modified to incorporate aptamers which have bind to prostate-specific membrane antigen (PSMA). The nanoparticles showed efficacy in PSMA positive LNCaP cells but not PSMA negative PC3 cells, exhibiting the selectivity of nanoparticles. Kolishetti et al. used a similar PEG–PLGA system to co-deliver docetaxel and cisplatin [129]. Similar to doxorubicin, cisplatin takes effect by inducing DNA damage [130]. These nanoparticles utilized the aptamer in [128] to target LNCaP prostate cancer cells. Apart from traditional polymeric nanoparticles, hydrogels composed of PEG–poly(ε-caprolactone-co-1,4,8-trioxa[4,6]spiro-9-undecanone) have been used for the controlled release of paclitaxel and doxorubicin to MCF-7 breast cancer cells in vitro and in vivo [131]. Baabur-Cohen et al. formed nanoparticles from paclitaxel and doxorubicin conjugated to polyglutamic acid (linear conjugate) or polyglycerol (dendritic conjugate) [132]. Both nanoparticles inhibited cell migration in MDA-MB-231 breast cancer cells. Inhibition of tumor growth and survival benefit in vivo was also shown for both types of nanoparticles. Recently, it has been shown that the benefits of using nanocarrier-mediated delivery of drugs may extend beyond relieving tumor burden, to restoring the changes in metabolic phenotype induced by breast cancer [133]. Metabonomics on mice bearing breast cancer xenograft revealed tumor-induced metabolic reprogramming in the liver, kidney, heart, and serum, which were partially reversed by the coadministration of doxorubicin and paclitaxel in soluble form. However, doxorubicin and paclitaxel delivery using PEG–PLGA most effectively reverted the metabolic changes.

#### 4.1.2. Other Paclitaxel Combinations

To combat resistance directly, co-delivery of paclitaxel and chemosensitizers (agents that sensitize cells to chemotherapy) have been attempted using nanoparticles. An added reason is that nanoparticle-bound drugs are less likely to effluxed out than free drugs [26]. Patil et al. loaded PLGA nanoparticles with paclitaxel and tariquidar (a P-glycoprotein inhibitor), which showed efficacy against JC, a mammary adenocarcinoma cell line, in vitro and in vivo [134]. The same system also showed efficacy against NCI/ADAR-RES cells, an ovarian adenocarcinoma cell line. Addition of biotin on the nanoparticle surface increased uptake by cells, leading to reduced tumor growth rate and prolonged survival in vivo. PEG–PCL micelles loaded with paclitaxel and ceramide decreased cell viability of SKOV3TR ovarian carcinoma cells and AML-12 hepatocytes compared to paclitaxel alone [135]. van Vlerken et al. coloaded PEG–PLGA nanoparticles with paclitaxel and ceramide to reduce cell viability in drug resistant SKOV3TR ovarian and MCF7TR breast cancer cells [136]. These nanoparticles were also the optimal performers in in vivo xenografts. Paclitaxel–ceramide nanoparticles induced most apoptosis (by TUNEL staining) and showed no toxicity. Wang and Ho loaded paclitaxel and combretastatin A4, an antiangiogenic drug, onto PEG–PLA nanoparticles [137]. The resulting nanoparticles showed efficacy both in vitro and in vivo, with maximum apoptosis and minimum toxicity in the latter. Further, it was also shown that the coadministration of paclitaxel and combretastatin reduced liver metastases in vivo, and dual drug-loaded nanoparticles prolonged survival in mice. PEG–PLGA nanoparticles loaded with paclitaxel and etoposide showed decreased viability, increased apoptosis, and higher proportion of cells undergoing G2/M phase arrest in osteosarcoma cells [138]. Paclitaxel and cisplatin carrying dendritic micelles exhibited efficacy against SKOV-3 ovarian cancer cells [139]. These micelles had a three-layer architecture, and complexation of cisplatin was facilitated by the introduction of carboxylic groups. Addition of cholic acids in the interior helped encapsulate paclitaxel better. The telodendrimeric micelles were taken up by the SKOV-3 cells leading to decreased cell viability, with a cisplatin: paclitaxel ratio of 2:1 exhibiting the most synergy. These nanoparticles also led to greatest growth suppression in vivo in mice bearing SKOV-3 xenografts. Extensive toxicity, bioavailability, and biodistribution studies were also carried out in vivo. Delivery of paclitaxel and cisplatin was attempted by Xiao et al. [140]. Cisplatin was loaded onto the micelles in a prodrug form and was released as active drug at low pH, such as in endosomes. Loading these drugs onto PEG–poly(lactide-co-2-methyl-2-carboxyl-propylene carbonate) micelles reduced the viability of SKOV-3 cells in vitro and reduced the growth rate of U14 cervical tumor xenografts. Polymeric micelles from triblock copolymer PEG–polyglutamic acid–polyphenylalanine were used by Desale et al. to obtain controlled and responsive drug release in the presence of proteolytic stimuli [141]. These micelles had a cross-linked intermediate layer of polyglutamic acid to ensure stability in the absence of proteolytic stimuli and were used to load paclitaxel and cisplatin. Dual drug loaded micelles exhibited synergistic toxicity in vitro and in vivo against A2780 ovarian cancer cells compared to single drugs. Biodistribution studies revealed higher accumulation in the tumor compared to free drug and increased levels of cleaved caspase-3, a marker of apoptosis. Recently, Wan et al. used polymeric micelles prepared from a triblock copolymer—poly(2-methyl-2-oxazoline-block-2-butyl-2-oxazoline-block-2-methyl-2-oxazoline)—to deliver paclitaxel and cisplatin [142]. The coloaded combination showed efficacy against cisplatin-resistant A278 ovarian cancer cells and multidrug-resistant LCC-6 breast cancer cells. Co-delivery of paclitaxel and cisplatin were also used to enhance efficiency of radiotherapy in lung cancer [143].

Zhao et al. used D-α-Tocopheryl polyethylene glycol succinate (TPGS), a derivative of vitamin E, to overcome P-glycoprotein-mediated drug resistance in ovarian cancer [144]. pH-sensitive micelles containing docetaxel and TPGS induced apoptosis in A2780/T ovarian cancer cells. Among surviving cell, majority were arrested in G2/M phase of the cell cycle. Finally, it was shown that TPGS reduced the activity of efflux pumps. Wang et al. used a similar concept to overcome multidrug resistance in non-small cell lung cancer cells [145]. Paclitaxel-5–fluorouracil–TPGS nanoparticles reduced the activity of efflux pumps, induced expression of the protein p53, and reduced cell viability in vitro. An alternative approach to overcome multidrug resistance is to facilitate apoptosis cells by reducing the levels of antiapoptotic proteins.

#### 4.1.3. Paclitaxel Combinations to Reduce Tumor Regrowth

Another solution has to been to deliver a ‘chemo-differentiative’ therapy. Paclitaxel acts as the cytotoxic agent here, while the ‘differentiative’ agent aims to irreversibly differentiate surviving cells into a quiescent state, incapable of initiating tumor regrowth [146]. Combination of Paclitaxel with Retinoic acid—a metabolite of Vitamin A that possesses differentiative properties—reduced resistance and relapse in liver and lung tumor xenografts [63]. Cylindrical micelles coloaded with retinoic acid and paclitaxel was found to be more cytotoxic in vitro. Significantly, the combination reduced regrowth of cells in vitro following drug treatment. In vivo, administration of dual drug-loaded micelles shrunk subcutaneous and intraperitoneal lung and liver tumors. Following cessation of treatment, retinoic acid–paclitaxel-treated tumors exhibited the slowest regrowth rate, while paclitaxel treated tumors grew at a rate comparable to untreated tumors. Mice treated with retinoic acid and paclitaxel exhibited prolonged survival. As retinoic acid was central to liver function, mice bearing orthotopic liver tumors were treated with the same combination. Similar to lung tumor xenografts, retinoic acid–paclitaxel-treated orthotopic tumors exhibited prolonged survival and had the smallest tumors when excised at the same endpoint. Cancer stem cells have also been postulated to mediated drug resistance and recurrence of tumors [147,148]. To combat the tumor renewing capability of cancer stem cells directly, Zhang et al. used salinomycin—shown to reduce breast cancer stem cells—in conjugation with paclitaxel [149]. These drugs were loaded onto PEG–PCL micelles. The surface of the micelles was octreotide-modified to facilitate binding to somatostatin receptors, which are overexpressed on breast cancer cells. Cellular uptake of these micelles by MCF-7 breast cancer cells was shown to be octreotide-mediated. Treatment with salinomycin reduced MCF-7 stem cells in vitro, and the dual drug combination exhibited maximum cytotoxicity in vitro. Combination treatment led to the smallest tumors in vivo, although it has to be noted that the two drugs were loaded into separate micelles and not coloaded. The excised tumors were disaggregated and stained for stem cell markers. Tumors that received micellar salinomycin had one-fourth the level of cancer stem cells as the untreated control. In a similar strategy, salinomycin and paclitaxel were delivered using HA-coated PLGA nanoparticles to reduce the fraction of breast cancer stem cells [150]. Yang et al. combined paclitaxel with curcumin to target breast cancer stem cells [151]. pH sensitive micelles coloaded with the drugs were taken up by MCF-7 breast cancer cells and led to a reduction in cell viability, percentage of stem cells, and mammosphere formation. These results were validated in vivo by demonstrating a reduction in tumor growth rate and fraction of cancer stem cells.

### 4.2. Other Doxorubicin Combinations

Doxorubicin in a hydrophilic anthracycline that induces apoptosis by causing DNA damage [152,153]. It exerts its DNA damaging effects via topoisomerase II inhibition and can also accelerate/cause apoptosis by generation of free radicals such as by production of hydrogen peroxide [154]. Like paclitaxel, efforts have been made to address doxorubicin drug resistance by co-delivering doxorubicin with other chemotherapeutics or sensitizers. Wu et al. synthesized poly acrylic acid-based nanogels that incorporated a disulfide bond, making the assembly sensitive to reducing stimuli, such as glutathione [155]. These nanoparticles were coloaded with doxorubicin and cisplatin. Multidrug-resistant MCF-7 breast cancer cells treated with these nanoparticles showed increased apoptosis and decreased survival in vitro. Combination treatment exhibited the slowest growth rate in vivo and showed reduced signs of toxicity (measured by body weight). Co-delivery of doxorubicin and Cyclosporin A reduced the growth rate of P388/ADR, a mouse lymphoma cell line [156]. Misra et al. coloaded PLGA nanoparticles with doxorubicin and curcumin, which showed efficacy against K562 leukemia cells [157]. These nanoparticles were taken up by the cells and led to a two-fold increased apoptosis compared to doxorubicin alone. In surviving cells, doxorubicin plus curcumin led to a G2/M arrest. Zhang et al. prepared pH-sensitive TPGS–poly amino ester loaded with curcumin and doxorubicin via solvent evaporation [158]. Mice bearing liver cancer xenografts shoed reduced tumor growth rate and angiogenesis after treatment with the dual drug nanoparticles. Ma et al. used HA–vitamin E–succinate micelles to deliver the same payload to breast cancer cells [159]. The doxorubicin–curcumin combination has also been used to overcome drug resistance in vitro and in vivo in breast cancer cells [160,161].

Drug carriers were established from polymer–drug conjugates comprised of the polymer N-(2-hydroxypropyl) methacrylamide and the drugs doxorubicin and gemcitabine [162]. These nanoparticles exhibited increased efficacy against Dunning AT1 rat prostrate carcinoma cells in vitro and in vivo. Tai et al. used doxorubicin and camptothecin-loaded nanoparticles, which were self-assembled from graft copolymers [163]. The copolymer had a PEG backbone with intermediate grafts of camptothecin–glutamate N–carboxyanhydride. The conjugated drug camptothecin is hydrophobic and drove the assembly in water to produce nanostructures. These nanoparticles showed high uptake in vitro with A549 lung cancer cells and slowed the growth of A549 xenografts in vivo. Duan et al. used poly(styrene-co-maleic anhydride) conjugated to doxorubicin to deliver a combination of doxorubicin and disulfiram (a P-glycoprotein inhibitor) to MCF-7/ADR breast cancer cells [164]. After proving cellular uptake and reduced cell viability in vitro, in vivo antitumor efficacy was demonstrated. While these cells were largely insensitive to doxorubicin alone, the addition of disulfiram increased efficacy of the chemotherapeutic and led to cell cycle arrest in the G2/M phase. PEG- poly(aspartate hydrazide) micelles containing doxorubicin and the PI3K inhibitor wortmannin demonstrated increased cytotoxicity against MCF-7 breast cancer cells [165]. pH sensitive hydrazone bond, which hydrolyzed under acidic environment and provided precise control over the release of encapsulated drugs.

Stepping up from co-delivery of two drugs, Liao et al. delivered doxorubicin, cisplatin, and camptothecin using polymeric nanoparticles [166]. These nanoparticles degraded by hydrolysis and photoirradiation and reduced the cell viability of OVCAR-3 ovarian cancer cells in vitro. Similar to the chemodifferentiative therapy of paclitaxel and retinoic acid discussed above [63], Sun et al. used PEG–PLA nanoparticles to codeliver doxorubicin and retinoic acid [167]. MDA-MB-231 breast cancer cells exhibit higher drug accumulation compared to the administration of soluble doxorubicin. In vitro, dual-treated cells displayed the lowest fraction of cancer stem cells and mRNA expression of treated cells revealed low levels of proteins that endow stem cell-like renewal capacity. In vivo, doxorubicin–retinoic acid-treated tumors exhibited reduced growth rates and had the lowest number of proliferating cells. A summary of the doxorubicin-based combinations described in this subsection is given in Table 2.

### 4.3. Drug–siRNA Combinations

The establishment of RNA interference (RNAi) as a viable means to disrupt gene expression has led to great interest in using nanocarriers to deliver siRNA (or shRNAs). Delivery of combinations of chemotherapeutics and siRNA have led to some success. PEG–PLA polymersomes with Bcl-xL siRNA and doxorubicin has been effective against gastric cancer cell lines [168]. Wang et al. loaded paclitaxel and Bcl2 siRNA onto nanoparticles self-assembled from P(MDS-co-CES) [169]. These nanoparticles reduced cell viability in MDA-MB-231 breast cancer cells. In an analogous study, Yu et al. delivered paclitaxel and Bcl-2 siRNA using poly(2-(dimethylamino)ethyl methacrylate)-block-poly(2-(diisopropylamino)ethyl methacrylate) polymeric micelles [170]. si-Bcl-2 reduced the expression of antiapoptotic Bcl-2 and led to reduced cell viability in vitro of lung cancer cells when co-delivered with paclitaxel compared to paclitaxel-scrambled siRNA system. Additionally, co-delivery of paclitaxel and interleukin-12 was also demonstrated. Interleukin-12 is an antitumor cytokine, and its presence led to reduced tumor growth in vivo in 4T1 breast cancer models than administration of paclitaxel-loaded nanoparticles. Yin et al. used poly[bis(2- hydroxylethyl)-disulfide-diacrylate-β-tetraethylenepentamine] (PBD)–PCL nanoparticles to deliver doxorubicin and shRNA against survivin, an antiapoptotic protein [171]. PBD–PCL inhibited activity of P-glycoprotein pumps. In vitro, the combination reduced cell growth in MCF-7/ADR cells, increasing apoptosis and reducing the number of dividing cells.

To overcome drug resistance in JC cells, Patil et al. loaded PLGA nanoparticles with paclitaxel and MDR1 siRNA [172]. This delivery system showed efficacy both in vitro and in vivo. Xiong and Lavasanifar used PEG–PCL micelles to deliver doxorubicin and MDR1 siRNA [173], while Pan et al. utilized PAMAM dendrimers to achieve the same feat [174]. In order to load siRNA and hydrophilic doxorubicin in nanoparticles with a hydrophobic core, the PCL block was modified to enable complexation with siRNA or drug. Further, the nanoparticle surface was decorated with RGD and cell penetrating TAT peptide. Delivery to drug-resistant MDA-MB-435 melanoma [175] cells resulted in reduced cell viability, while delivery of doxorubicin alone had little success. The addition of integrin-binding RGD peptide on the surface further increased efficacy of delivery. In vivo, RGD functionalized micelles exhibited higher accumulation in tumor and lesser presence in the liver and spleen than nonfunctionalized micelles. PEG–PLGA nanoparticles delivered cisplatin and anti-luciferase siRNA to luciferase-expressing HeLa cells [176]. Following successful silencing of luciferase activity, siRNA-mediated suppression of DNA translesion synthesis genes was attempted. This pathway is essential for cellular response to cisplatin-induced DNA adduct formation, and its silencing markedly increased in vitro sensitivity of LNCaP prostate cancer cells to cisplatin. In vivo, silencing of the same pathway prevented tumor growth and increased survival in LNCaP tumor xenograft-bearing mice. Other nanoparticle platforms such as dendrimers have been utilized to codeliver drugs and siRNA. Kaneshiro et al. provided a proof-of-concept by delivering doxorubicin and anti-luciferase siRNA to silence luciferase activity in U-87 glioblastoma cells [177].

### 4.4. Additional Combinations

Methotrexate and retinoic acid coloaded into PAMAM dendrimers displayed increased cytotoxicity in vitro [178]. Miao et al. delivered cisplatin and gemcitabine to UMUC3 bladder cancer cells using PLGA nanoparticles [179]. Drug loading was achieved by solvent displacement method. Combination therapy exhibited the lowest IC50 in vitro and led to the smallest tumors in vivo. Combination nanoparticles exhibited synergy (as indicated by CI <1) at a gemcitabine: cisplatin ratio of ~5.5:1, while the combination in free-form achieved synergy only at high concentrations. Observed in vivo efficacy was supported by higher apoptosis and reduced proliferation in tumors treated with gemcitabine plus cisplatin. Delivery of another platinum drug—oxaliplatin—was attempted along with 5-fluorouracil [180]. PLGA/poly(3-hydroxybutyrate-co-3-hydroxyvalerate acid) nanoparticles loaded with drugs were prepared using a double emulsion method and used against HT29 and CT26 colon cancer cells. Ni et al. used a biotin-/lactobionic acid-modified PEG–PLGA–PEG nanoparticles to deliver 5-fluorouracil and curcumin to liver cancer cells [181]. Decreased tumor burden and increased apoptosis was demonstrated in mice bearing HepG2 xenografts.

Coadministration of vincristine and verapamil by PLGA nanoparticles showed growth inhibition in MCF7/ADR cells; breast cancer cell that were resistant to vincristine [182]. Coencapsulation of tamoxifen (used regularly in clinic for the treatment of breast cancer) and quercetin in PLGA nanoparticles reduced viability of MCF-7 breast cancer cells and exhibited high uptake by Caco-2 colorectal cancer cells [183]. In vivo, tamoxifen–quercetin nanoparticles shrunk DMBA-induced breast tumors and prolonged survival. Tamoxifen–quercetin nanoparticles also reduced the levels of the matrix metalloproteinases MMP-2 and MMP-9 in the plasma. A summary of the combinations described in the last two subsections is given in Table 3. PLGA–PEG nanoparticles were used to deliver a combination of gemcitabine and betulinic acid to PANC1, a pancreatic cancer cell line [184]. Increased toxicity and apoptosis were demonstrated in vitro, followed by reduction of tumor volume in vivo.

Beside the above-mentioned examples, delivery of drugs can also be attempted using other types of nanoparticles such as core–shell [185] and Janus [186,187,188].

## 5. Targeting Specific Oncogenic Pathways

**Figure 2:**
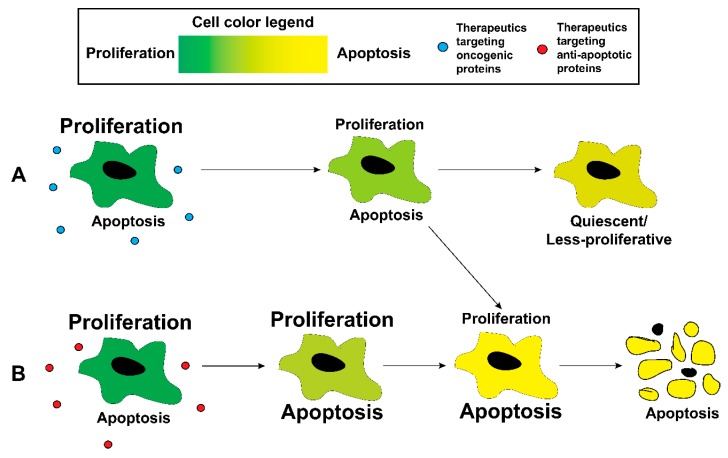
Treatment of proliferative cancer cells with either (**A**) therapeutics targeting oncogenic pathways or (**B**) therapeutics targeting antiapoptotic proteins can increase the probability of the cell undergoing apoptosis or undergoing quiescence.

While the delivery of combination chemotherapy using nanoparticles has been a success—much like combination chemotherapy in clinic itself—these efforts still leave a significant room for improvement. Nanoparticle delivery of combination chemotherapy has been performed in vivo without invoking the off-target effects of cytotoxic chemotherapy [136,137,140]. However, the continuous administration of these drugs—unlike drug administration for a fixed time-point study—could eventually lead to toxicity and the occurrence of off-target effects as mentioned in Section 1.2. As was the case with combination chemotherapy, inspiration can be drawn from current practices in clinic. The solution to overcoming cytotoxicity associated with chemotherapy was the introduction and administration of targeted chemotherapy, that aimed to suppress certain specific pathways. However, the administration of targeted chemotherapeutics has been plagued with similar problems as cytotoxic chemotherapy (Section 1.2). Better delivery, either alone as adjuvant and neoadjuvant treatments, or in combination with other cytotoxic chemotherapy might alleviate this cytotoxicity. The rest of this review will focus on key pathways to target—oncogenic pathways to inhibit or antioncogenic pathways to augment—as well as nanoparticle-based efforts to achieve precisely this. The discussion falls mainly into two categories: inhibiting oncogenic pathways (Figure 2A) or inhibiting antiapoptotic proteins to facilitate apoptosis (Figure 2B). It has to be noted that only studies that use polymer-based nanoparticles are reported here. There are a multitude of investigation performed with other nanoparticles, including lipid-based and iron oxide nanoparticles, that are beyond the scope of this review. A summary of the highlighted studies can be found in Table 4.

### 5.1. Growth Factor Receptors, MAPK, and PI3K/Akt Signaling Cascades

Excessive growth/proliferation is one of the ‘hallmarks of cancer’ [1,189,190], and these growth signals are often mediated by receptors on the cell surface such as HER2 and EGFR [191,192]. The overexpression of these receptors by cancer cells presents an opportunity to specifically deliver drugs using actively targeting nanoparticles. An example of this was provided by Bahadur et al., who used nanoparticles with HER2 targeting ligands on the surface [193]. Nanoparticles were synthesized from the polymer PEG–poly[2-(pyridin-2-yldisulfanyl)], and camptothecin was conjugated to the polymer via disulfide bonds. The anti-HER2 antibody Herceptin was conjugated to the surface after nanoparticle formation. Herceptin conferred selectivity of targeting to cells that overexpressed HER2, while the presence of disulfide bonds made the system labile to reducing conditions such as the presence of glutathione or dithiothreitol. Compared to nanoparticles with no targeting ligand, herceptin nanoparticles reduced cell viability in HER2 positive HCT-116 cells, but not in HER2 negative KB cells.

Acharya et al. used EGFR targeting PLGA nanoparticles to deliver rapamycin to MCF-7 breast cancer cells [194]. Targeted nanoparticles displayed higher uptake in vitro and possessed more cytotoxicity than nontargeted nanoparticles. Delivery of rapamycin inhibited mTOR, inducing cell cycle arrest and apoptosis. Other studies targeting EGFR expression on the surface of breast [195] and lung [196] cancer cells have also been reported. Cui et al. delivered microRNA-7 (miR-7) to ovarian cancer cells in order to suppress the EGFR/ERK pathway [197]. Encapsulation of miR-7 in PEG–PLGA-PLL nanoparticles enhanced stability of the microRNA in the serum and led to increased transfection efficiency in HO-8910PM ovarian cancer cells. Incorporation of paclitaxel alongside miR-7 led to increased apoptosis and reduced ERK activation. The in vitro observations and mechanisms were validated in an in vivo mouse model bearing ovarian cancer xenografts.

Receptor-based growth signals are often transduced by the Mitogen-activated protein kinase (MAPK) pathway [198]. Deregulations that hyperactivate the MAPK cascade is common across various types of cancer [199] and plays an important role behind high proliferation rates observed in cancer cells. Basu et al. targeted the MAPK pathway using PD98059, an inhibitor of MEK1 [200]. After conjugating PD98059 to PLGA, PLGA-PD98059 was blended with PEG–PLGA, and nanoparticles were formed by the emulsion–solvent evaporation method. These nanoparticles reduced the proliferation rate of MDA-MB-231 breast cancer and B16/F10 melanoma cells in vitro. In vivo validation was performed in mice bearing B16/F10 xenografts. PD98059 nanoparticles plus intraperitoneal administration of cisplatin led to the smallest tumors, with induction of apoptosis and minimal MAPK activation observed in tissue sections. Chen et al. used PLGA nanoparticles to deliver sorafenib, a RAF inhibitor, and AZD6244, a MEK inhibitor [201]. The nanoparticle surface was lipid-based and modified with a peptide to target CXCR4. In vitro, the nanoparticles reduced cell viability and MAPK pathway activity in HCC cells. Upregulation of BIM, a proapoptotic protein, and suppression of angiogenesis was also observed. Targeted nanoparticles displayed increased uptake in orthotopic HCC model in vivo. As observed in vitro, the nanoparticles decreased both MAPK pathway activity and angiogenesis in tumors. Finally, suppression of PD-L1 expression was also reported, along with increased cytotoxic T cell accumulation. Sorafenib, in combination with doxorubicin, was delivered with PLGA nanoparticles to reduce the viability of HT-29 colon cancer cells [202].

Another growth signaling pathway that is commonly dysregulated in cancers is the phosphatidylinositol-3-kinase (PI3K)–Akt pathway [203]. Harfouche et al. used PLGA nanoparticles loaded with LY294002 to target the PI3K pathway in melanoma and breast cancer cells [204]. These nanoparticles reduced cell viability and the levels of phospho-AKT, downstream effector of PI3K, in MDA-MB-231 and B16/F10 cells in vitro. In vivo in a zebrafish model, these models demonstrated inhibition of angiogenesis in both tumor models. Lu et al. used TGX-221, another PI3K inhibitor, to inhibit phosphorylation of the downstream protein Akt [205]. Poly(3-hydroxybutyrate)–TGX-221 nanoparticles were prepared by ultrasonication. Inhibition of PI3K reduced the growth rate of PC3 prostate cancer cells and reduction on phospho-Akt levels was confirmed by Western blot. Gholizadeh et al. used a PI3K and mTOR inhibitor—Dactolisib (BEZ)—to reduce inflammation levels in endothelial cells [206]. PLGA nanoparticles loaded with BEZ were surface-modified to target E-selectin via an E-selectin antibody and showed preferential uptake to nontargeted nanoparticles. BEZ nanoparticles reduced HUVEC cell migration when measured by a scratch wound assay. Reduced levels of phospho-PI3K, phospho-S6 (downstream of mTORC1), and phospho-Akt, with targeted nanoparticles demonstrating increased suppression than control nontargeted nanoparticles.

### 5.2. STAT3 Signaling

The Janus kinase (JAK)–signal transducer and activator of transcription (STAT) signaling pathway is known to promote inflammation, proliferation, and invasion in cancer [207,208]. Much like the MAPK pathway, the JAK–STAT pathway also integrates numerous signals and regulates the transcription of various oncogenic proteins. Of the members of the STAT family, STAT3 in particular has been associated with facilitating and sustaining tumorigenesis [207,208]. STAT3 also modulates the recruitment of stromal cells such as immune cells and promotes immune evasion [208]. Hence, targeting STAT3 has become a major endeavor in cancer therapy [209] and efforts to reduce STAT3 signaling using nanoparticles have been attempted. Curcumin, a polyphenol derived from turmeric, has been shown to inhibit STAT3 signaling in multiple cancers [210,211]. Lim et al. used curcumin loaded polymeric nanoparticles to reduce proliferation and survival of embryonal tumor and glioblastoma cells [212]. Reduced level of phospho-STAT3 was confirmed using Western blot, along with reductions in IGF-1R level and hedgehog pathway. This correlated with reduced proliferation, increased apoptosis, and decreased stemness/self-renewal capacity. In addition to STAT3, curcumin-loaded nanoparticles have been shown to inhibit other oncogenic proteins as well. Yallapu et al. examined the effects of curcumin on prostate cancer cells using PLGA–curcumin nanoparticles [213]. After demonstrating reduced growth and clonogenicity in vitro in three prostate cancer cell lines, the antitumorigenic effects of PLGA–curcumin nanoparticles were demonstrated in vivo. Reduced levels for STAT3, downstream phospho-Akt, β-catenin, androgen receptor, the antiapoptotic proteins Bcl-xL and Mcl-1, and microRNAs deregulated in prostate cancer. A similar drug delivery system was used with success in ovarian cancer [214]. Molavi et al. used PLGA nanoparticles to deliver JSI-124, a selective STAT3 inhibitor, to B16 melanoma cells [215]. Treated cells exhibited reduced cell viability and a reduction in levels of phospho-STAT3. Additionally, it was also shown that the nanoparticles reduced phospho-STAT3 levels in dendritic cells and induce proliferation of T cells in vivo. Recently, another STAT3 inhibitor—S3I-1757—was utilized by Huang et al. to demonstrate efficacy against multiple myeloma cells that overexpress CD38 on the surface [216]. PEG-poly(α-benzyl carboxylate-ε-caprolactone) nanoparticles were decorated with anti-CD38 antibodies to target U266 myeloma cells. Targeted nanoparticles were more cytotoxic than the nontargeted ones and caused a greater reduction in levels of phospho-STAT3. These observations were validated in vivo, with CD38 targeting nanoparticles leading to a 75% reduction in tumor size compared to nontargeting nanoparticles. As was observed in vitro, CD38-targeting nanoparticles reduced phospho-STAT3 levels more efficiently than nontargeting nanoparticles.

Besides small molecule inhibitors to decrease STAT3 activation, another approach has been to deliver siRNAs to reduce STAT3 expression. Alshamsan et al. reduced phopsho-STAT3 levels in B16 cells by delivering STAT3 siRNA using PLGA nanoparticles [217]. Release and uptake studies revealed that the nanoparticles were internalized by the cells in 4 to 8 h, while the encapsulated siRNA was released in 10 days. Western blots for phopsho-STAT3 showed reduced levels compared to scrambled siRNA controls. The incorporation of polyethylenimine (PEI)–stearic acid to the polymers reduced the degradation of STAT3 siRNA, leading to better efficacy in vitro. Similar to [215], STAT3 silencing led to the return of dendritic cell maturation and activity. Su et al. showed that incorporation of STAT3 siRNA to paclitaxel can increase efficacy of treatment in vitro and potentially sensitize paclitaxel-resistant A549 lung cancer cells to the chemotherapeutic [218]. Using PLGA nanoparticles, the delivery of siRNA reduced levels of phospho-STAT3 and lead to increased apoptosis. Das et al. used a similar PEI–PLGA nanoparticle system to deliver STAT3 siRNA [219]. STAT3 siRNA reduced cell viability and increased apoptosis and led to an increase cell cycle arrest in surviving cells at the G0/G1 phase. A similar PLGA-based system was used to deliver STAT3 and TLR7 siRNA to dendritic cells [220]. The particles exhibited higher uptake by dendritic cells, leading to reduced STAT3 mRNA levels in vitro. A reduction in levels of STAT3 was accompanied by increased secretion of cytokines TNF-α and IL-12p70 and increased cytotoxic T cell activity. Finally, when mice were challenged with EG7-OVA lymphomas, nanoparticle-treated mice displayed the slowest tumor growth rate, indicating more efficient stimulation of the immune system was by the nanoparticles.

### 5.3. Facilitating Apoptosis

Delivery of siRNAs targeting members of the antiapoptotic families of protein have been mentioned in Section 4.3, including siRNAs against Bcl-2 [169,170], Bcl-xL [168], and survivin [171]. Numerous other attempts that successfully target Bcl-2 have been reported, including additional siRNA mediated depletions [221,222,223,224] and utilization of a peptide to turn Bcl-2 proapoptotic from antiapoptotic [225]. NuBCP-9 peptide interacts with Bcl-2 and induces a conformation change that turns it proapoptotic. Kumar et al. encapsulated NuBCP-9 peptide in PEG–PLA and PEG–polypropylene glycol–PEG–PLA nanoparticles [225]. Increasing the PEG chain length was found to enhance peptide loading, while the chain lengths of both PEG and PLA controlled the release profile. The peptide-loaded nanoparticles induced apoptosis in MCF-7 breast and HepG2 liver cancer cells in vitro, which was backed up by pronounced levels of cleaved caspase-3 after 48 h. In vivo, NuBCP-9 nanoparticles induced regression of mammary adenocarcinomas and prolonged survival in treated mice. Notably, administration of the peptide in soluble form provided marginal benefit in tumor growth and survival. More recently, Wang et al. delivered a Bcl-2 converting gene—Nur77—and paclitaxel to liver cancer cells using PHB-PDMAEMA micelles [226].

Bcl-xL, another antiapoptotic protein of the Bcl family, has been a popular target. Kim et al. enhanced cell death in prostate cancer by delivering Bcl-xL shRNA in addition to doxorubicin [227]. The PEG–PEI copolymer was conjugated to aptamers targeting PSMA and doxorubicin was intercalated into the aptamer. As in Zhang et al. [128], the nanoparticles showed efficacy in PSMA positive LNCaP cells but not PSMA negative PC3 cells. LNCaP cells that received shRNA showed much lower cell viability after 48 h of incubation. However, the viability was similar irrespective of the presence of shRNA for time scales less than a day, indicating that it takes at least two days for the shRNA to take effect. Finally, delivery using nanoparticles led to higher cytotoxicity than administration of the drug in soluble form and shRNA (complexed with lipofectamine). In an analogous approach to target breast cancer, Ebrahimian et al. used PLGA–PEI nanoparticles to deliver doxorubicin and Bcl-xL shRNA [228]. Ayatollahi et al. used aptamer conjugated dendrimers to deliver Bcl-xL shRNA to lung cancer [229]. The nucleolin-targeting AS1411 aptamer was chosen as it is expressed by various cancer cells including the A549, the lung cancer cells used in this study. AS1411-dendrimers loaded with Bcl-xL shRNA reduced the Bcl-xL protein levels compared to scrambled shRNA. Consistent with the loss of an antiapoptotic protein, cells treated with Bcl-xL shRNA showed increased apoptosis. Additionally, nanoparticles with aptamer on the surface performed better than nanoparticles without aptamers.

Shen et al. co-delivered survivin shRNA and paclitaxel using mixed polymeric micelles to non-small cell lung cancer cells in an attempt to overcome drug resistance [230]. The micelles displayed uptake in both A549 and paclitaxel-resistant A549/T cells. While there was no difference in the cytotoxicity of soluble paclitaxel vs. nanoparticle paclitaxel in A549 cells, nanoparticle paclitaxel was more effective than soluble form in A549/T cells. The addition of survivin shRNA did not change survival in A549 cells but did so in A549/T cells. This was validated by increased apoptosis in A549/T cells upon treatment with paclitaxel-shRNA nanoparticle. In vitro observations were successfully translated in vivo. Mice bearing A549/T xenografts exhibited lowest tumor growth rate with paclitaxel-shRNA nanoparticles and showed no toxicity compared to saline controls. The delivery of survivin siRNA and paclitaxel was demonstrated by Jin et al. [231]. Paclitaxel was incorporated into the core of the PEI-PLA nanoparticles during self-assembly, followed by siRNA complexation. PEG–poly(L-aspartic acid) was then coated on the surface to yield the final nanoparticles. Silencing of survivin gene and reduced cell viability was demonstrated in A549 lung cancer cells in vitro. Tumor shrinkage and enhanced survival was also demonstrated in mice bearing A549 subcutaneous xenografts. Another strategy to target survivin was explored by Wang et al., who delivered the miRNA miR-542-3p (an inhibitor of survivin) along with doxorubicin [232]. PEI–PLGA nanoparticles with HA on the surface were used to target breast cancer cells overexpressing the receptor CD44. Doxorubicin was also utilized, this time in conjunction with survivin siRNA in metastatic lung cancer by Xu et al. [233].

p53 is a master regulator of apoptosis and delivery of the p53 gene was attempted by Chen et al. [234]. A poly(2-dimethylaminoethyl methacrylate) polycationic brush was used for the co-delivery of doxorubicin and p53 (pDNA) to MCF-7 breast cancer cells. Maximum growth inhibition was observed for combination treatment, hinting at synergy between the two therapeutics. Similarly, PEI–PCL nanoparticles have been utilized to deliver doxorubicin and p53 plasmid DNA to cervical and liver cancer cells [235].

### 5.4. Wnt/β-Catenin Pathway

The Wnt/β-catenin pathway is critical for maintain stemness. It regulates a number of normal and neoplastic processes, and is known to be dysregulated in various cancers [236]. The attenuation of β-catenin signaling by PLGA–curcumin nanoparticles has been described in prostate [213] and ovarian [214] cancers (Section 5.2). Poly(propylene imine) dendrimers surface modified with maltotriose was found to inhibit the Wnt pathway in chronic lymphocytic leukemia cells [237]. Microarray analysis performed on treated and untreated MEC-1 leukemia cells revealed downregulation of seven members of the Wnt/β-catenin pathway from a total of nineteen (> 33%).

### 5.5. Hedgehog Signaling

The hedgehog signaling pathway is a major developmental pathway which is also commonly dysregulated in cancers [238]. Chenna et al. used PEG–PLGA nanoparticles to encapsulate HPI-1, a hedgehog pathway inhibitor, and target medulloblastomas and orthotopic pancreatic cancer [239]. HPI-1 nanoparticles reduced tumor growth rates in both in vivo models, leading to smallest tumors when treated with the Hedgehog inhibitor. Reduced levels of hedgehog signaling were confirmed in both models and minimal toxicity was observed in mice compared to the vehicle control. Xu et al. showed that tumors positive for Patched-1, a member of the hedgehog family, were associated with higher recurrence rate in HCC [240]. The same drug delivery system as [239] was also used to reduce HCC growth and metastasis in vivo. Reduced levels of CD133 (marker of cancer stemness) and MAPK pathway activity was also shown. Besides cancer, Hedgehog inhibitors have also been delivered using PEG–poly(carbonate-co-lactide) nanocarriers to attenuate liver fibrosis [241].

### 5.6. Hypoxia

Inadequate blood supply to tumors often lead to low oxygen levels (or hypoxia) in certain parts of tumors [242]. One of the main transcription factors induced by hypoxia is the hypoxia-inducible factor HIF-1α, which has been implicated in chemoresistance and metastasis. Hence, there has been a general interest in targeting HIF-1α to slow down malignant progression of tumors. Nanoparticle based attempts to achieve this goal have focused on using RNAi to disrupt HIF-1α expression. Liu et al. used PEG–poly(2-aminoethylethylene phosphate) and PEG–PCL micelles to deliver HIF-1α siRNA to prostate cancers [243]. Reduction in HIF-1α levels was accompanied by a decrease in proliferation and migration of PC3 cells as well as decreased angiogenesis by HUVEC cells in vitro. Codelivery of HIF-1α siRNA with doxorubicin reduced tumor growth rate in vivo while recapitulating other in vitro observations. Similarly, polymer–lipid nanoparticles have been used to deliver HIF-1α siRNA and gemcitabine to treat pancreatic cancers [244]. Lipid–polymer nanoparticles produced a greater suppression in tumor growth rate than polymer nanoparticles and exhibited better tumor accumulation. Efficacy of HIF-1α inhibition was also demonstrated in orthotopic pancreatic cancer model. Aside from cancer, PLGA nanoparticles have delivered HIF-1α siRNA to reduce angiogenesis in the eye to attenuate choroidal neovascularization [245].

### 5.7. Targeting Myc

Myc is a potent oncogene whose expression is highly regulated in normal cells [246]. Located at the intersection of several oncogenic pathways, elevated Myc expression is reported in many cancers and results in overactive proliferation. Tangudu et al. prepared nanoparticles from polyglycidal methacrylate–polyethyleneimine embedded with iron oxide cores to deliver Myc shRNA [247]. Silencing of Myc was proven in MDA-MB-231 breast cancer cells in vitro. shRNA nanoparticles controlled tumor growth rate in a spontaneous breast cancer model, doubling survival time in mice. Similar effects were observed in an induced model of colorectal cancer and reduced expression of Myc was confirmed by Western blotting. shRNA treated mice showed better tissue morphology and reduced staining for Myc by IHC.

## 6. Conclusions and Perspectives on Future Directions

Nanoparticles hold immense promise and has shown success in vitro and in vivo. But, this success has translated poorly into clinics. While there are exceptions, they are too few and mostly restricted to liposomal nanoparticles such as Doxil [248]. Polymeric nanoparticles are gaining prominence in clinic and could realize the potential of nanotechnology in cancer treatment. The most promising breakthroughs, however, may arrive from hybrid polymer–lipid nanoparticles [58,249,250,251,252]. Additionally, prior experience in clinic and literature, both hint at the fact that combination and targeted chemotherapy are the paths forward.

Inherent heterogeneity among cancer cells posits that the administration of a chemotherapy puts a selective pressure on the cells, encouraging the emergence of drug-resistant cells [26]. This is one of the main reasons behind the eventual relapse among patients that is observed in clinic. Combination chemotherapy has been the solution in clinic, and a vast body of literature (Section 4) supports that the same might hold true with nanocarriers. In all the work cited in Section 4, the administration of combination of drugs provided greater benefit than the single drugs. Additionally, delivery by nanocarriers can lead to longer circulation times, precise control over drug release, and improved pharmacokinetics. In many cases, synergy was observed between the drugs. Of course, it has to be noted that not all drug combinations will be synergetic or additive. Combinations will exist that are sub-additive or even antagonistic; these are less likely to be reported. One popular theme among drug combinations is complementarity in mode of action. Combinations such as taxanes and DNA damage-inducing agents or combinations of different DNA damage-inducing agents have been one of the main focuses of this paper. Combinations of chemotherapeutics with siRNAs was also briefly listed. However, to fully comprehend the mechanism of action of the combination, detailed investigations into the underlying cell biology is warranted. This is particularly true for combination treatments over single drug treatments. The cause of any synergy between two drugs might be due to a cross-talk between their main pathways/targets. A modification in signaling or the pharmacokinetics of drugs either by (1) the introduction of nanoparticles and/or (2) improved nanocarrier-based delivery may be the difference between success and failure in clinic ([63] and the success of Doxil and Abraxane).

Comprehension of the underlying biology could be a significant advantage during chemotherapy, as activation of alternate pathways in response to administered treatment may drive resistance [26]. However, reliance on alternate pathways for acquisition of resistance can be exploited by the addition of respective inhibitors to the treatment regimen. This could greatly diminish the emergence of resistant cells and a similar concept is at the heart of the principle of ‘synthetic lethality’ [253]. A prominent example is the use of PARP inhibitors to treat BRCA deficient tumors. BRCA and PARP are both involved in DNA damage repair and to avoid excessive DNA damage, tumor cells are dependent on PARP—much like how cancer cells are dependent on specific resistance mechanisms during chemotherapy. Hence, the inhibition of PARP in BRCA deficient tumors leads to cell death [254], and administration of PARP inhibitors to BRCA deficient tumors is a standard practice in clinic. Understanding the underlying biology of combination therapies also opens a door to the incorporation of targeted chemotherapeutics (Section 5) into chemotherapy regimens. Additionally, how nanoparticles themselves and the administration of drugs by nanoparticles alter the biology of the cell is not well understood. A deeper understanding of cancer biology could lead to therapeutics better capable of distinguishing between cancer and normal cells. This could particularly be significant in the case of personalized therapies. Personalized treatments bank on the knowledge of how a particular patient’s tumor can be targeted given its biology. Not only could deeper investigations into inhibition of oncogenic signaling pathways provide a means to deliver this therapy effectively, knowing how delivery systems affect biology can help better predict responses to nanoparticle-based treatments.

Overall, as cancer treatments steadily move towards factoring-in underlying biology, corresponding advances are required in our understanding of how nanoparticles affect and are affected by the various signaling pathways. It is plausible that optimal chemotherapeutic regimens in the near future could be a combination of cytotoxic/targeted chemotherapeutics and specific pathway inhibitors delivered by nanoparticles.

## Figures and Tables

**Table 1:** Summary of studies using polymeric nanoparticle systems to deliver paclitaxel-based combination therapies.

Polymer Used	Cancer Type	Drugs Delivered	Reference
PEG-poly(l-glutamic acid)-b-poly(l-lysine)	Lung	Paclitaxel, doxorubicin	[125]
PEG–PLGA	Lung, liver, melanoma	Paclitaxel, doxorubicin	[126]
PEG–PCL	Breast	Docetaxel, doxorubicin	[127]
PEG–PLGA	Prostate	Docetaxel, doxorubicin	[128]
PEG–PLGA	Prostate	Docetaxel, cisplatin	[129]
PEG–poly(ε-caprolactone-co-1,4,8-trioxa[4.6]spiro-9-undecanone)	Breast	Paclitaxel, doxorubicin	[131]
Polyglutamic acid, polyglycerol	Breast	Paclitaxel, doxorubicin	[132]
PEG–PLGA	Breast	Paclitaxel, doxorubicin	[133]
PLGA	Breast, ovarian	Paclitaxel, tariquidar	[134]
PEG–PCL	Ovarian, liver	Paclitaxel, ceramide	[135]
PEG–PLGA	Breast, ovarian	Paclitaxel, ceramide	[136]
PEG–PLA	Lung, melanoma	Paclitaxel, combretastatin A4	[137]
PEG–PLGA	Bone	Paclitaxel, etoposide	[138]
PEG-cholic acid telodendrimer	Ovarian	Paclitaxel, cisplatin	[139]
PEG–poly(lactide-co-2-methyl-2-carboxyl-propylene carbonate)	Ovarian	Paclitaxel, cisplatin	[140]
PEG–polyglutamic acid–polyphenylalanine	Ovarian	Paclitaxel, cisplatin	[141]
Poly(MeOx-BuOx-MeOx)	Ovarian, breast	Paclitaxel, cisplatin	[142]
PLGA–PEG	Lung	Paclitaxel, cisplatin	[143]
TPGS-poly β-amino ester	Ovarian	Docetaxel, TPGS	[144]
Paclitaxel-TPGS-5-Fluorouracil	Lung	Paclitaxel, 5-fluorouracil, TPGS	[145]
PEG–PCL	Liver, lung	Paclitaxel, retinoic acid	[63]
PEG–PCL	Breast	Paclitaxel, salinomycin	[149]
HA-PLGA	Breast	Paclitaxel, salinomycin	[150]
PEG–benzoic imine-poly(γ-benzyl-l-aspartate)-b-poly(1-vinylimidazole)	Breast	Paclitaxel, curcumin	[151]

**Table 2:** Summary of studies using polymeric nanoparticle systems to deliver doxorubicin-based combination therapies.

Polymer Used	Cancer Type	Drugs Delivered	Reference
Poly acrylic acid	Breast	Doxorubicin, cisplatin	[155]
Polyalkylcyanoacrylate	Lymphoma	Doxorubicin, cyclosporin A	[156]
PLGA	Leukemia	Doxorubicin, curcumin	[157]
TPGS-PAE	Liver	Doxorubicin, curcumin	[158]
HA–vitamin E–succinate	Breast	Doxorubicin, curcumin	[159]
Poly(curcumin-dithiodipropionic acid)–PEG–biotin	Breast	Doxorubicin, curcumin	[160]
PEG–PLGA-poly(L-glutamic acid)	Breast	Doxorubicin, curcumin	[161]
Poly(N-(2-hydroxypropyl) methacrylamide)	Prostate	Doxorubicin, gemcitabine	[162]
PEG-poly(*γ*-camptothecin–glutamate N–carboxyanhydride)	Lung	Doxorubicin, camptothecin	[163]
Poly(styrene-co-maleic anhydride)	Breast	Doxorubicin, disulfiram	[164]
PEG–poly(aspartate hydrazide)	Breast	Doxorubicin, wortmannin	[165]
PEG macromonomers	Ovarian	Doxorubicin, cisplatin, and camptothecin	[166]
PEG–PLA	Breast	Doxorubicin, retinoic acid	[167]

**Table 3:** Summary of studies using polymeric nanoparticle systems to deliver other combination therapies.

Polymer Used	Cancer Type	Drugs Delivered	Reference
PEG–PLA	Gastric	Doxorubicin, Bcl-xL siRNA	[168]
P(MDS-co-CES)	Breast	Paclitaxel, Bcl-2 siRNA,	[169]
Poly(2-(dimethylamino)ethyl methacrylate)– poly(2-(diisopropylamino)ethyl methacrylate)	Lung	Paclitaxel, Bcl-2 siRNA,	[170]
PBD–PCL	Breast	Doxorubicin, Survivin shRNA,	[171]
PLGA	Breast	Paclitaxel, MDR1 siRNA	[172]
PEG–PCL	Melanoma	Doxorubicin, MDR1 siRNA	[173]
PAMAM dendrimer	Ovarian, breast	Doxorubicin, MDR1 siRNA	[174]
PEG–PLGA	Cervical, prostate	Cisplatin, REV1 and REV3L siRNA	[176]
Poly(l-lysine)–PEG dendrimers	Glioblastoma	Doxorubicin, anti-luciferase siRNA	[177]
PAMAM dendrimer	Cervical	Methotrexate, retinoic acid	[178]
PLGA	Bladder	Cisplatin, gemcitabine	[179]
PLGA/ poly(3-hydroxybutyrate-co-3-hydroxyvalerate acid)	Colon	Oxaliplatin, 5-fluorouracil	[180]
PEG–PLGA–PEG	Liver	5-fluorouracil, curcumin	[181]
PLGA	Breast	Vincristine, verapamil	[182]
PLGA	Breast	Tamoxifen, quercetin	[183]
PLGA–PEG	Pancreatic	Gemcitabine, betulinic acid	[184]

**Table 4:** Summary of studies using polymeric nanoparticle systems to target pathways for cancer therapy.

Protein/ Pathway	Cancer Type	Drug Delivery System Used	Drug/Therapeutic(s) Delivered	Reference
HER2	Colon	PEG–poly[2-(pyridin-2-yldisulfanyl)]	Camptothecin	[193]
EGFR	Breast	PLGA	Rapamycin	[194]
EGFR	Breast	PLGA–PEG	Curcumin	[195]
EGFR	Lung	PLGA	Docetaxel	[196]
EGFR	Ovarian	PEG–PLGA-PLL	miR-7, paclitaxel	[197]
MEK1/MAPK	Breast, melanoma	PLGA	PD98059, cisplatin	[200]
MEK, RAF/MAPK	Liver	PLGA	Sorafenib, AZD6244	[201]
RAF/MAPK	Colon	PLGA/PEG–PLGA	Sorafenib, doxorubicin	[202]
PI3K/Akt	Breast, melanoma	PLGA	LY294002	[204]
PI3K/Akt	Prostate	Poly(3-hydroxybutyrate) drug conjugate	TGX-221	[205]
PI3K, mTOR	N/A (Endothelial cells)	PLGA	Dactolisib	[206]
STAT3	Embryonal, glioblastoma	Poly (N-isopropylacrylamide – vinylpyrrolidone–acrylic acid)	Curcumin	[212]
STAT3, β-Catenin	Prostate	PLGA	Curcumin	[213]
STAT3, β-catenin	Ovarian	PLGA	Curcumin	[214]
STAT3	Melanoma	PLGA	JSI-124	[215]
STAT3	Myeloma	PEG-poly(α-benzyl carboxylate-ε-caprolactone)	S3I-1757	[216]
STAT3	Melanoma	PLGA	STAT3 siRNA	[217]
STAT3	Lung	PLGA, PEI–stearic acid	STAT3 siRNA, paclitaxel	[218]
STAT3	Lung	PEI–PLGA	STAT3 siRNA	[219]
STAT3	N/A (Dendritic cells)	PLGA	STAT3 siRNA, TLR7 siRNA	[220]
Bcl-2	Breast	P(MDS-co-CES)	Bcl-2 siRNA, paclitaxel	[169]
Bcl-2	Lung	Poly(2-(dimethylamino)ethyl methacrylate)-poly(2-(diisopropylamino)ethyl methacrylate)	Bcl-2 siRNA, paclitaxel	[170]
Bcl-2	Lung, cervical, breast	P(MDS-co-CES)	Bcl-2 siRNA	[221]
Bcl-2	Breast	Core–shell polymeric nanoparticles	Bcl-2 siRNA	[222]
Bcl-2	Neuroblastoma	PEG–PEI	Bcl-2 siRNA	[223]
Bcl-2	Liver	PEG-PLL	Bcl-2 siRNA, Doxorubicin	[224]
Bcl-2	Liver	PEG–PLA and PEG-PPG-PEG–PLA	NuBCP-9 peptide	[225]
Bcl-2	Liver	PHB-PDMAEMA	Bcl-2 siRNA, paclitaxel	[226]
Bcl-xL	Gastric	PEG–PLA	Bcl-xL siRNA, doxorubicin	[168]
Bcl-xL	Prostate	PEG–PEI	Bcl-xL siRNA, doxorubicin	[227]
Bcl-xL	Breast	PLGA–PEI	Bcl-xL siRNA, doxorubicin	[228]
Bcl-xL	Lung	PAMAM dendrimers	Bcl-xL siRNA	[229]
Survivin	Breast	PBD–PCL	Survivin shRNA, doxorubicin	[171]
Survivin	Lung	P85-PEI/TPGS	Survivin shRNA, paclitaxel	[230]
Survivin	Lung	(PEG-PAsp)(PEI-PLA)	Survivin siRNA, paclitaxel	[231]
Survivin	Breast	HA/PEI–PLGA	miR-542-3p, doxorubicin	[232]
Survivin	Lung	PEI- 3-Maleimidopropionic acid hydrazide	Survivin siRNA, doxorubicin	[233]
p53	Breast	PDMAEMA	p53 gene, doxorubicin	[234]
p53	Cervical, liver	PEI–PCL	p53 plasmid DNA, doxorubicin	[235]
Wnt/β-Catenin	Chronic lymphocytic leukemia	Poly(propylene imine)	Maltotriose	[237]
Hedgehog	Pancreatic, medulloblastomas	PEG–PLGA	HPI-1	[239]
Hedgehog	Liver	PEG–PLGA	HPI-1	[240]
Hedgehog	N/A (Liver fibrosis)	PEG-poly(carbonate-co-lactide)	Vismodegib, rosiglitazone	[241]
HIF-1α/ hypoxia	Prostate	PEG-poly(2-aminoethylethylene phosphate), PEG–PCL	HIF-1α siRNA, doxorubicin	[243]
HIF-1α/ hypoxia	Pancreatic	Polymer–lipid hybrid	HIF-1α siRNA, gemcitabine	[244]
HIF-1α/ hypoxia	N/A (Choroidal neovascularization)	PLGA	HIF-1α siRNA	[245]
Myc	Breast, colorectal	polyglycidal methacrylate – polyethyleneimine	Myc shRNA	[247]

## Abbreviations

EGFR	Epidermal growth factor receptor
EPR	Enhanced Permeation and Retention
HA	Hyaluronic acid
HCC	Hepatocellular carcinoma
HER2	Human epidermal growth factor receptor 2
HIF	Hypoxia-inducible factor
IC50	Half maximal inhibitory concentration
MAPK	Mitogen-activated protein kinase
MEK	Mitogen-activated protein kinase
MTD	Maximum tolerated dose
PAMAM	Polyamidoamine
PARP	Poly (ADP-ribose) polymerase
PCL	Poly(ε-caprolactone)
PEI	Polyethylenimine
PEG	Polyethylene glycol
PI3K	Phosphatidylinositol-3-kinase
PLA	Poly-L-lactic acid
PLGA	Poly(lactic-co-glycolic acid)
PLL	Poly-L-lysine
PSMA	Prostate-specific membrane antigen
RAF	Rapidly Accelerated Fibrosarcoma
RTK	Receptor tyrosine kinase
siRNA	Small interfering Ribonucleic Acid
shRNA	Small hairpin Ribonucleic Acid
STAT	Signal transducer and activator of transcription
TPGS	D-α-Tocopheryl polyethylene glycol succinate

## References

[1] Hanahan D., Weinberg R.A. (2011). Hallmarks of cancer: The next generation. Cell.

[2] Siegel R.L., Miller K.D., Jemal A. (2019). Cancer statistics, 2019. CA Cancer J. Clin..

[3] Bray F., Ferlay J., Soerjomataram I., Siegel R.L., Torre L.A., Jemal A. (2018). Global cancer statistics 2018: GLOBOCAN estimates of incidence and mortality worldwide for 36 cancers in 185 countries. CA Cancer J. Clin..

[4] DeSantis C.E., Lin C.C., Mariotto A.B., Siegel R.L., Stein K.D., Kramer J.L., Alteri R., Robbins A.S., Jemal A. (2014). Cancer treatment and survivorship statistics, 2014. CA Cancer J. Clin..

[5] Greaves M., Maley C.C. (2012). Clonal evolution in cancer. Nature.

[6] Marzo I., Naval J. (2013). Antimitotic drugs in cancer chemotherapy: Promises and pitfalls. Biochem. Pharmacol..

[7] Feld R., Sridhar S.S., Shepherd F.A., Mackay J.A., Evans W.K. (2006). Use of the Epidermal Growth Factor Receptor Inhibitors Gefitinib and Erlotinib in the Treatment of Non-small Cell Lung Cancer: A Systematic Review. J. Thorac. Oncol..

[8] Leamon C.P., Reddy J.A. (2004). Folate-targeted chemotherapy. Adv. Drug. Deliv. Rev..

[9] Druker B.J. (2002). STI571 (Gleevec^TM^) as a paradigm for cancer therapy. Trends Mol. Med..

[10] Flaherty K.T., Puzanov I., Kim K.B., Ribas A., McArthur G.A., Sosman J.A., O’Dwyer P.J., Lee R.J., Grippo J.F., Nolop K. (2010). Inhibition of Mutated, Activated BRAF in Metastatic Melanoma. N. Engl. J. Med..

[11] Groopman J.E., Itri L.M. (1999). Chemotherapy-Induced Anemia in Adults: Incidence and Treatment. JNCI J. Natl. Cancer Inst..

[12] Falleti M.G., Sanfilippo A., Maruff P., Weih L., Phillips K.-A. (2005). The nature and severity of cognitive impairment associated with adjuvant chemotherapy in women with breast cancer: A meta-analysis of the current literature. Brain Cogn..

[13] Partridge A.H., Burstein H.J., Winer E.P. (2001). Side Effects of Chemotherapy and Combined Chemohormonal Therapy in Women With Early-Stage Breast Cancer. JNCI Monogr..

[14] Sitzia J., Huggins L. (1998). Side effects of cyclophosphamide, methotrexate, and 5-fluorouracil (CMF) chemotherapy for breast cancer. Cancer Pract..

[15] Lemieux J., Maunsell E., Provencher L. (2008). Chemotherapy-induced alopecia and effects on quality of life among women with breast cancer: A literature review. Psychooncology.

[16] Chidambaram M., Manavalan R., Kathiresan K. (2011). Nanotherapeutics to Overcome Conventional Cancer Chemotherapy Limitations. J. Pharm. Pharm. Sci..

[17] Lotfi-Jam K., Carey M., Jefford M., Schofield P., Charleson C., Aranda S. (2008). Nonpharmacologic strategies for managing common chemotherapy adverse effects: A systematic review. J. Clin. Oncol..

[18] Penn I., Starzl T.E. (1973). Immunosuppression and cancer. Transplant. Proc..

[19] Simbre V.C., Duffy S.A., Dadlani G.H., Miller T.L., Lipshultz S.E. (2005). Cardiotoxicity of Cancer Chemotherapy. Pediatr. Drugs.

[20] Monsuez J.-J., Charniot J.-C., Vignat N., Artigou J.-Y. (2010). Cardiac side-effects of cancer chemotherapy. Int. J. Cardiol..

[21] King P.D., Perry M.C. (2001). Hepatotoxicity of chemotherapy. Oncologist.

[22] Ries F., Klastersky J. (1986). Nephrotoxicity Induced by Cancer Chemotherapy With Special Emphasis on Cisplatin Toxicity. Am. J. Kidney Dis..

[23] Robert C., Soria J.-C., Spatz A., Le Cesne A., Malka D., Pautier P., Wechsler J., Lhomme C., Escudier B., Boige V. (2005). Cutaneous side-effects of kinase inhibitors and blocking antibodies. Lancet Oncol..

[24] Frei E., Canellos G.P. (1980). Dose: A critical factor in cancer chemotherapy. Am. J. Med..

[25] Tredan O., Galmarini C.M., Patel K., Tannock I.F. (2007). Drug Resistance and the Solid Tumor Microenvironment. JNCI J. Natl. Cancer Inst..

[26] Markman J.L., Rekechenetskiy A., Holler E., Ljubimova J.Y. (2013). Nanomedicine therapeutic approaches to overcome cancer drug resistance. Adv. Drug Deliv. Rev..

[27] Bokemeyer C., Oechsle K., Honecker F., Mayer F., Hartmann J.T., Waller C.F., Bohlke I., Kollmannsberger C. (2007). Combination chemotherapy with gemcitabine, oxaliplatin, and paclitaxel in patients with cisplatin-refractory or multiply relapsed germ-cell tumors: A study of the German Testicular Cancer Study Group. Ann. Oncol..

[28] Frei E., Eder J.P., Kufe D.W., Pollock R.E., Weichselbaum R.R., Bast R.C., Gansler T.S., Holland J.F., Frei E.M. (2003). Combination Chemotherapy. Holland-Frei Cancer Medicine.

[29] Chen Q., Xia H.-W., Ge X.-J., Zhang Y.-C., Tang Q.-L., Bi F. (2013). Serum miR-19a Predicts Resistance to FOLFOX Chemotherapy in Advanced Colorectal Cancer Cases. Asian Pac. J. Cancer Prev..

[30] Chua W., Goldstein D., Lee C.K., Dhillon H., Michael M., Mitchell P., Clarke S.J., Iacopetta B. (2009). Molecular markers of response and toxicity to FOLFOX chemotherapy in metastatic colorectal cancer. Br. J. Cancer.

[31] Lee S., Oh S.Y., Kim S.H., Lee J.H., Kim M.C., Kim K.H., Kim H.-J. (2013). Prognostic significance of neutrophil lymphocyte ratio and platelet lymphocyte ratio in advanced gastric cancer patients treated with FOLFOX chemotherapy. BMC Cancer.

[32] Canellos G.P., Anderson J.R., Propert K.J., Nissen N., Cooper M.R., Henderson E.S., Green M.R., Gottlieb A., Peterson B.A. (1992). Chemotherapy of Advanced Hodgkin’s Disease with MOPP, ABVD, or MOPP Alternating with ABVD. N. Engl. J. Med..

[33] Diehl V., Franklin J., Pfreundschuh M., Lathan B., Paulus U., Hasenclever D., Tesch H., Herrmann R., Dörken B., Müller-Hermelink H.-K. (2003). Standard and Increased-Dose BEACOPP Chemotherapy Compared with COPP-ABVD for Advanced Hodgkin’s Disease. N. Engl. J. Med..

[34] Diehl V., Sieber M., Rüffer U., Lathan B., Hasenclever D., Pfreundschuh M., Loeffler M., Lieberz D., Koch P., Adler M. (1997). BEACOPP: An intensified chemotherapy regimen in advanced Hodgkin’s disease. Ann. Oncol..

[35] Cummings F.J., Gelman R., Horton J. (1985). Comparison of CAF versus CMFP in metastatic breast cancer: Analysis of prognostic factors. J. Clin. Oncol..

[36] Ozols R.F., Hogan W.M., Ostchega Y., Young R.C. (1983). MVP (mitomycin, vinblastine, and progesterone): A second-line regimen in ovarian cancer with a high incidence of pulmonary toxicity. Cancer Treat. Rep..

[37] Smith I.E., Powles T.J. (1993). MMM (mitomycin/mitoxantrone/methotrexate): An effective new regimen in the treatment of metastatic breast cancer. Oncology.

[38] Fang J., Nakamura H., Maeda H. (2011). The EPR effect: Unique features of tumor blood vessels for drug delivery, factors involved, and limitations and augmentation of the effect. Adv. Drug Deliv. Rev..

[39] Hu Q., Sun W., Wang C., Gu Z. (2016). Recent advances of cocktail chemotherapy by combination drug delivery systems. Adv. Drug Deliv. Rev..

[40] Folkman J. (1995). Angiogenesis in cancer, vascular, rheumatoid and other disease. Nat. Med..

[41] Suzuki M., Takahashi T., Sato T. (1987). Medial regression and its functional significance in tumor-supplying host arteries. A morphometric study of hepatic arteries in human livers with hepatocellular carcinoma. Cancer.

[42] Torchilin V. (2011). Tumor delivery of macromolecular drugs based on the EPR effect. Adv. Drug Deliv. Rev..

[43] Matsumura Y., Maeda H. (1986). A new concept for macromolecular therapeutics in cancer chemotherapy: Mechanism of tumoritropic accumulation of proteins and the antitumor agent smancs. Cancer Res..

[44] Seki T., Fang J., Maeda H. (2009). Enhanced delivery of macromolecular antitumor drugs to tumors by nitroglycerin application. Cancer Sci..

[45] Acharya S., Sahoo S.K. (2011). PLGA nanoparticles containing various anticancer agents and tumour delivery by EPR effect. Adv. Drug Deliv. Rev..

[46] Maeda H., Wu J., Sawa T., Matsumura Y., Hori K. (2000). Tumor vascular permeability and the EPR effect in macromolecular therapeutics: A review. J. Control. Release.

[47] De Jong W.H., Borm P.J.A. (2008). Drug delivery and nanoparticles:applications and hazards. Int. J. Nanomed..

[48] Kipp J.E. (2004). The role of solid nanoparticle technology in the parenteral delivery of poorly water-soluble drugs. Int. J. Pharm..

[49] Moghimi S.M., Hunter A.C., Murray J.C. (2001). Long-circulating and target-specific nanoparticles: Theory to practice. Pharmacol. Rev..

[50] Yoo J.-W., Chambers E., Mitragotri S. (2010). Factors that control the circulation time of nanoparticles in blood: Challenges, solutions and future prospects. Curr. Pharm. Des..

[51] Bazak R., Houri M., El Achy S., Kamel S., Refaat T. (2015). Cancer active targeting by nanoparticles: A comprehensive review of literature. J. Cancer Res. Clin. Oncol..

[52] Pearce T.R., Shroff K., Kokkoli E. (2012). Peptide Targeted Lipid Nanoparticles for Anticancer Drug Delivery. Adv. Mater..

[53] Xu S., Cui F., Huang D., Zhang D., Zhu A., Sun X., Cao Y., Ding S., Wang Y., Gao E. (2019). PD-L1 monoclonal antibody-conjugated nanoparticles enhance drug delivery level and chemotherapy efficacy in gastric cancer cells. Int. J. Nanomed..

[54] Wang Q., Zhong Y., Liu W., Wang Z., Gu L., Li X., Zheng J., Du H., Zhong Z., Xie F. (2019). Enhanced chemotherapeutic efficacy of the low-dose doxorubicin in breast cancer via nanoparticle delivery system crosslinked hyaluronic acid. Drug Deliv..

[55] Lin C.-J., Kuan C.-H., Wang L.-W., Wu H.-C., Chen Y., Chang C.-W., Huang R.-Y., Wang T.-W. (2016). Integrated self-assembling drug delivery system possessing dual responsive and active targeting for orthotopic ovarian cancer theranostics. Biomaterials.

[56] Alexis F., Pridgen E., Molnar L.K., Farokhzad O.C. (2008). Factors Affecting the Clearance and Biodistribution of Polymeric Nanoparticles. Mol. Pharm..

[57] Stolnik S., Illum L., Davis S.S. (1995). Long circulating microparticulate drug carriers. Adv. Drug Deliv. Rev..

[58] Zhang L., Chan J.M., Gu F.X., Rhee J.-W., Wang A.Z., Radovic-Moreno A.F., Alexis F., Langer R., Farokhzad O.C. (2008). Self-Assembled Lipid−Polymer Hybrid Nanoparticles: A Robust Drug Delivery Platform. ACS Nano..

[59] Mattheolabakis G., Rigas B., Constantinides P.P. (2012). Nanodelivery strategies in cancer chemotherapy: Biological rationale and pharmaceutical perspectives. Nanomedicine.

[60] Meng F., Zhong Z., Feijen J. (2009). Stimuli-Responsive Polymersomes for Programmed Drug Delivery. Biomacromolecules.

[61] Li S., Byrne B., Welsh J., Palmer A.F. (2007). Self-Assembled Poly(butadiene)-b-poly(ethylene oxide) Polymersomes as Paclitaxel Carriers. Biotechnol. Prog..

[62] Li Y.Y., Cunin F., Link J.R., Gao T., Betts R.E., Reiver S.H., Chin V., Bhatia S.N., Sailor M.J. (2003). Polymer replicas of photonic porous silicon for sensing and drug delivery applications. Science.

[63] Nair P.R., Alvey C., Jin X., Irianto J., Ivanovska I., Discher D.E. (2018). Filomicelles Deliver a Chemo-Differentiation Combination of Paclitaxel and Retinoic Acid That Durably Represses Carcinomas in Liver to Prolong Survival. Bioconjug. Chem..

[64] Krause H.-J., Schwarz A., Rohdewald P. (1985). Polylactic acid nanoparticles, a colloidal drug delivery system for lipophilic drugs. Int. J. Pharm..

[65] Makadia H.K., Siegel S.J., Makadia H.K., Siegel S.J. (2011). Poly Lactic-co-Glycolic Acid (PLGA) as Biodegradable Controlled Drug Delivery Carrier. Polymers.

[66] Kazunori K., Glenn S.K., Masayuki Y., Teruo O., Yasuhisa S. (1993). Block copolymer micelles as vehicles for drug delivery. J. Control. Release.

[67] Nair P.R., Karthick S., Spinler K.R., Vakili M.R., Lavasanifar A., Discher D.E. (2016). Filomicelles from aromatic diblock copolymers increase paclitaxel-induced tumor cell death and aneuploidy compared with aliphatic copolymers. Nanomedicine.

[68] Singh R., Lillard J.W. (2009). Nanoparticle-based targeted drug delivery. Exp. Mol. Pathol..

[69] Herrero-Vanrell R., Rincón A.C., Alonso M., Reboto V., Molina-Martinez I.T., Rodríguez-Cabello J.C. (2005). Self-assembled particles of an elastin-like polymer as vehicles for controlled drug release. J. Control Release.

[70] Panyam J., Labhasetwar V. (2003). Biodegradable nanoparticles for drug and gene delivery to cells and tissue. Adv. Drug Deliv. Rev..

[71] Rajagopal K., Mahmud A., Christian D.A., Pajerowski J.D., Brown A.E.X., Loverde S.M., Discher D.E. (2010). Curvature-Coupled Hydration of Semicrystalline Polymer Amphiphiles Yields flexible Worm Micelles but Favors Rigid Vesicles: Polycaprolactone-Based Block Copolymers. Macromolecules.

[72] Peppas N.A., Duncan R., Wnek G.E., Hoffman A.S., Gao G.H., Kim S.W., Lee D.S., Hadjiargyrou M., Touitou E., Ainbinder D. (2014). Highly cited research articles in Journal of Controlled Release: Commentaries and perspectives by authors. J. Control Release.

[73] Discher D.E., Ahmed F. (2006). Polymersomes. Annu. Rev. Biomed. Eng..

[74] Akamatsu K., Shimada M., Tsuruoka T., Nawafune H., Fujii S., Nakamura Y. (2010). Synthesis of pH-Responsive Nanocomposite Microgels with Size-Controlled Gold Nanoparticles from Ion-Doped, Lightly Cross-Linked Poly(vinylpyridine). Langmuir.

[75] Oltra N.S., Nair P., Discher D.E. (2014). From Stealthy Polymersomes and Filomicelles to “Self” Peptide-Nanoparticles for Cancer Therapy. Annu. Rev. Chem. Biomol. Eng..

[76] Cuomo F., Lopez F., Piludu M., Miguel M.G., Lindman B., Ceglie A. (2015). Release of small hydrophilic molecules from polyelectrolyte capsules: Effect of the wall thickness. J. Colloid Interface Sci..

[77] Leong K.W., Brott B.C., Langer R. (1985). Bioerodible polyanhydrides as drug-carrier matrices. I: Characterization, degradation, and release characteristics. J. Biomed. Mater. Res..

[78] Middleton J.C., Tipton A.J. (2000). Synthetic biodegradable polymers as orthopedic devices. Biomaterials.

[79] Gao W., Chan J.M., Farokhzad O.C. (2010). pH-Responsive Nanoparticles for Drug Delivery. Mol. Pharm..

[80] Borchert U., Lipprandt U., Bilang M., Kimpfler A., Rank A., Peschka-Süss R., Schubertm R., Lindner P., Förster S. (2006). pH-Induced Release from P2VP−PEO Block Copolymer Vesicles. Langmuir.

[81] Chécot F., Lecommandoux S., Klok H.-A., Gnanou Y. (2003). From supramolecular polymersomes to stimuli-responsive nano-capsules based on poly(diene-b-peptide) diblock copolymers. Eur. Phys. J. E.

[82] Cho H., Bae J., Garripelli V.K., Anderson J.M., Jun H.-W., Jo S. (2012). Redox-sensitive polymeric nanoparticles for drug delivery. Chem. Commun..

[83] Cerritelli S., Velluto D., Hubbell J.A. (2007). PEG-SS-PPS: Reduction-Sensitive Disulfide Block Copolymer Vesicles for Intracellular Drug Delivery. Biomacromolecules.

[84] Napoli A., Boerakker M.J., Tirelli N., Nolte R.J.M., Sommerdijk N.A.J.M., Hubbell J.A. (2004). Glucose-oxidase based self-destructing polymeric vesicles. Langmuir.

[85] Mansour A.M., Drevs J., Esser N., Hamada F.M., Badary O.A., Unger C., Schubert R., Lindner P., Förster S. (2003). A new approach for the treatment of malignant melanoma: Enhanced antitumor efficacy of an albumin-binding doxorubicin prodrug that is cleaved by matrix metalloproteinase 2. Cancer Res..

[86] Pan J., Li P.-J., Wang Y., Chang L., Wan D., Wang H. (2018). Active targeted drug delivery of MMP-2 sensitive polymeric nanoparticles. Chem. Commun..

[87] Yan B., Boyer J.-C., Branda N.R., Zhao Y. (2011). Near-Infrared Light-Triggered Dissociation of Block Copolymer Micelles Using Upconverting Nanoparticles. J. Am. Chem. Soc..

[88] Feng H., Zhao Y., Pelletier M., Dan Y., Zhao Y. (2009). Synthesis of photo- and pH-responsive composite nanoparticles using a two-step controlled radical polymerization method. Polymer.

[89] Li D., Jones G.L., Dunlap J.R., Hua F., Zhao B. (2006). Thermosensitive hairy hybrid nanoparticles synthesized by surface-initiated atom transfer radical polymerization. Langmuir.

[90] Cuomo F., Cofelice M., Venditti F., Ceglie A., Miguel M., Lindman B., Lopez F. (2018). In-vitro digestion of curcumin loaded chitosan-coated liposomes. Colloids Surfaces B Biointerfaces.

[91] Szatrowski T.P., Nathan C.F. (1991). Production of large amounts of hydrogen peroxide by human tumor cells. Cancer Res..

[92] Ganta S., Devalapally H., Shahiwala A., Amiji M. (2008). A review of stimuli-responsive nanocarriers for drug and gene delivery. J. Control Release.

[93] Sultana S., Khan M.R., Kumar M., Kumar S., Ali M. (2013). Nanoparticles-mediated drug delivery approaches for cancer targeting: A review. J. Drug Target..

[94] Jacob J., Haponiuk J.T., Thomas S., Gopi S. (2018). Biopolymer based nanomaterials in drug delivery systems: A review. Mater. Today Chem..

[95] Vauthier C., Bouchemal K. (2009). Methods for the Preparation and Manufacture of Polymeric Nanoparticles. Pharm. Res..

[96] Amoabediny G., Haghiralsadat F., Naderinezhad S., Helder M.N., Akhoundi Kharanaghi E., Mohammadnejad Arough J., Zandieh-Doulabi B. (2018). Overview of preparation methods of polymeric and lipid-based (niosome, solid lipid, liposome) nanoparticles: A comprehensive review. Int. J. Polym. Mater. Polym. Biomater..

[97] Cuomo F., Ceglie A., De Leonardis A., Lopez F., Cuomo F., Ceglie A., De Leonardis A., Lopez F. (2019). Polymer Capsules for Enzymatic Catalysis in Confined Environments. Catalysts.

[98] Gaitzsch J., Huang X., Voit B. (2016). Engineering Functional Polymer Capsules toward Smart Nanoreactors. Chem. Rev..

[99] Rao J.P., Geckeler K.E. (2011). Polymer nanoparticles: Preparation techniques and size-control parameters. Prog. Polym. Sci..

[100] Gradishar W.J., Tjulandin S., Davidson N., Shaw H., Desai N., Bhar P., Hawkins M., O’Shaughnessy J. (2005). Phase III Trial of Nanoparticle Albumin-Bound Paclitaxel Compared With Polyethylated Castor Oil–Based Paclitaxel in Women With Breast Cancer. J. Clin. Oncol..

[101] Wang F., Porter M., Konstantopoulos A., Zhang P., Cui H. (2017). Preclinical development of drug delivery systems for paclitaxel-based cancer chemotherapy. J. Control Release.

[102] Sofou S. (2007). Surface-active liposomes for targeted cancer therapy. Nanomedicine.

[103] Ali A., Ahmed S. (2018). A review on chitosan and its nanocomposites in drug delivery. Int. J. Biol. Macromol..

[104] Elgadir M.A., Uddin M.S., Ferdosh S., Adam A., Chowdhury A.J.K., Sarker M.Z.I. (2015). Impact of chitosan composites and chitosan nanoparticle composites on various drug delivery systems: A review. J. Food Drug Anal..

[105] Ching S.H., Bansal N., Bhandari B. (2017). Alginate gel particles–A review of production techniques and physical properties. Crit. Rev. Food Sci. Nutr..

[106] Paques J.P., van der Linden E., van Rijn C.J.M., Sagis L.M.C. (2014). Preparation methods of alginate nanoparticles. Adv. Colloid Interface Sci..

[107] Kita-Tokarczyk K., Grumelard J., Haefele T., Meier W. (2005). Block copolymer vesicles—using concepts from polymer chemistry to mimic biomembranes. Polymer.

[108] Yu D.-G., Zheng X.-L., Yang Y., Li X.-Y., Williams G.R., Zhao M. (2019). Immediate release of helicid from nanoparticles produced by modified coaxial electrospraying. Appl. Surf. Sci..

[109] Mayer L.D., Bally M.B., Cullis P.R. (1986). Uptake of adriamycin into large unilamellar vesicles in response to a pH gradient. Biochim. Biophys. Acta Biomembr..

[110] Bonnemain B. (1998). Superparamagnetic Agents in Magnetic Resonance Imaging: Physicochemical Characteristics and Clinical Applications A Review. J. Drug Target..

[111] Juliano R.L., Alahari S., Yoo H., Kole R., Cho M. (1999). Antisense Pharmacodynamics: Critical Issues in the Transport and Delivery of Antisense Oligonucleotides. Pharm. Res..

[112] Fattal E., Vauthier C., Aynie I., Nakada Y., Lambert G., Malvy C., Couvreur P. (1998). Biodegradable polyalkylcyanoacrylate nanoparticles for the delivery of oligonucleotides. J. Control Release.

[113] Devita V.T., Young R.C., Canellos G.P. (1975). Combination versus single agent chemotherapy: A review of the basis for selection of drug treatment of cancer. Cancer.

[114] Woodcock J., Griffin J.P., Behrman R.E. (2011). Development of Novel Combination Therapies. N. Engl. J. Med..

[115] Hu C.-M.J., Zhang L. (2012). Nanoparticle-based combination therapy toward overcoming drug resistance in cancer. Biochem. Pharmacol..

[116] Jia J., Zhu F., Ma X., Cao Z.W., Li Y.X., Chen Y.Z. (2009). Mechanisms of drug combinations: Interaction and network perspectives. Nat. Rev. Drug Discov..

[117] Wall M.E., Wani M.C. (1995). Camptothecin and taxol: Discovery to clinic—Thirteenth Bruce F. Cain Memorial Award Lecture. Cancer Res..

[118] Wall M.E. (1998). Camptothecin and taxol: Discovery to clinic. Med. Res. Rev..

[119] Long B.H., Fairchild C.R. (1994). Paclitaxel inhibits progression of mitotic cells to G1 phase by interference with spindle formation without affecting other microtubule functions during anaphase and telephase. Cancer Res..

[120] Jordan M.A., Wendell K., Gardiner S., Derry W.B., Copp H., Wilson L. (1996). Mitotic block induced in HeLa cells by low concentrations of paclitaxel (Taxol) results in abnormal mitotic exit and apoptotic cell death. Cancer Res..

[121] Cochran M.C., Eisenbrey J., Ouma R.O., Soulen M., Wheatley M.A. (2011). Doxorubicin and paclitaxel loaded microbubbles for ultrasound triggered drug delivery. Int. J. Pharm..

[122] Dong X., Mattingly C.A., Tseng M.T., Cho M.J., Liu Y., Adams V.R., Mumper R.J. (2009). Doxorubicin and Paclitaxel-Loaded Lipid-Based Nanoparticles Overcome Multidrug Resistance by Inhibiting P-Glycoprotein and Depleting ATP. Cancer Res..

[123] Cui Y., Xu Q., Chow P.K.-H., Wang D., Wang C.-H. (2013). Transferrin-conjugated magnetic silica PLGA nanoparticles loaded with doxorubicin and paclitaxel for brain glioma treatment. Biomaterials.

[124] Wang Y., Ma S., Xie Z., Zhang H. (2014). A synergistic combination therapy with paclitaxel and doxorubicin loaded micellar nanoparticles. Colloids Surfaces B Biointerfaces.

[125] Lv S., Tang Z., Li M., Lin J., Song W., Liu H., Huang Y., Zhang Y., Chen X. (2014). Co-delivery of doxorubicin and paclitaxel by PEG-polypeptide nanovehicle for the treatment of non-small cell lung cancer. Biomaterials.

[126] Wang H., Zhao Y., Wu Y., Hu Y., Nan K., Nie G., Chen H. (2011). Enhanced anti-tumor efficacy by co-delivery of doxorubicin and paclitaxel with amphiphilic methoxy PEG-PLGA copolymer nanoparticles. Biomaterials.

[127] Wu J., Zhang H., Hu X., Liu R., Jiang W., Li Z., Luan Y. (2018). Reduction-sensitive mixed micelles assembled from amphiphilic prodrugs for self-codelivery of DOX and DTX with synergistic cancer therapy. Colloids Surfaces B Biointerfaces.

[128] Zhang L., Radovic-Moreno A.F., Alexis F., Gu F.X., Basto P.A., Bagalkot V., Jon S., Langer R.S., Farokhzad O.C. (2007). Co-Delivery of Hydrophobic and Hydrophilic Drugs from Nanoparticle–Aptamer Bioconjugates. ChemMedChem.

[129] Kolishetti N., Dhar S., Valencia P.M., Lin L.Q., Karnik R., Lippard S.J., Langer R., Farokhzad O.C. (2010). Engineering of self-assembled nanoparticle platform for precisely controlled combination drug therapy. Proc. Natl. Acad. Sci. USA.

[130] Siddik Z.H. (2003). Cisplatin: Mode of cytotoxic action and molecular basis of resistance. Oncogene.

[131] Wang W., Song H., Zhang J., Li P., Li C., Wang C., Kong D., Zhao Q. (2015). An injectable, thermosensitive and multicompartment hydrogel for simultaneous encapsulation and independent release of a drug cocktail as an effective combination therapy platform. J. Control Release.

[132] Baabur-Cohen H., Vossen L.I., Krüger H.R., Eldar-boock A., Yeini E., Landa-Rouben N., Tiram G., Wedepohl S., Markovsky E., Leor J. (2017). In vivo comparative study of distinct polymeric architectures bearing a combination of paclitaxel and doxorubicin at a synergistic ratio. J. Control Release.

[133] Hu Y., Zhu X., Zhao R., Wang J., Song Y., Nie G., Tang H., Wang Y. (2018). Doxorubicin and paclitaxel carried by methoxy poly(ethylene glycol)-poly(lactide-co-glycolide) is superior than traditional drug-delivery methods. Nanomedicine.

[134] Patil Y., Sadhukha T., Ma L., Panyam J. (2009). Nanoparticle-mediated simultaneous and targeted delivery of paclitaxel and tariquidar overcomes tumor drug resistance. J. Control Release.

[135] van Vlerken L.E., Duan Z., Seiden M.V., Amiji M.M. (2007). Modulation of Intracellular Ceramide Using Polymeric Nanoparticles to Overcome Multidrug Resistance in Cancer. Cancer Res..

[136] van Vlerken L.E., Duan Z., Little S.R., Seiden M.V., Amiji M.M. (2010). Augmentation of Therapeutic Efficacy in Drug-Resistant Tumor Models Using Ceramide Coadministration in Temporal-Controlled Polymer-Blend Nanoparticle Delivery Systems. AAPS J..

[137] Wang Z., Ho P.C. (2010). A nanocapsular combinatorial sequential drug delivery system for antiangiogenesis and anticancer activities. Biomaterials.

[138] Wang B., Yu X.-C., Xu S.-F., Xu M. (2015). Paclitaxel and etoposide co-loaded polymeric nanoparticles for the effective combination therapy against human osteosarcoma. J. Nanobiotechnol..

[139] Cai L., Xu G., Shi C., Guo D., Wang X., Luo J. (2015). Telodendrimer nanocarrier for co-delivery of paclitaxel and cisplatin: A synergistic combination nanotherapy for ovarian cancer treatment. Biomaterials.

[140] Xiao H., Song H., Yang Q., Cai H., Qi R., Yan L., Liu S., Zheng Y., Huang Y., Liu T. (2012). A prodrug strategy to deliver cisplatin(IV) and paclitaxel in nanomicelles to improve efficacy and tolerance. Biomaterials.

[141] Desale S.S., Cohen S.M., Zhao Y., Kabanov A.V., Bronich T.K. (2013). Biodegradable hybrid polymer micelles for combination drug therapy in ovarian cancer. J. Control Release.

[142] Wan X., Beaudoin J.J., Vinod N., Min Y., Makita N., Bludau H., Jordan R., Wang A., Sokolsky M., Kabanov A.V. (2019). Co-delivery of paclitaxel and cisplatin in poly(2-oxazoline) polymeric micelles: Implications for drug loading, release, pharmacokinetics and outcome of ovarian and breast cancer treatments. Biomaterials.

[143] Tian J., Min Y., Rodgers Z., Au K.M., Hagan C.T., Zhang M., Roche K., Yang F., Wagner K., Wang A.Z. (2017). Co-delivery of paclitaxel and cisplatin with biocompatible PLGA–PEG nanoparticles enhances chemoradiotherapy in non-small cell lung cancer models. J. Mater. Chem. B.

[144] Zhao S., Tan S., Guo Y., Huang J., Chu M., Liu H., Zhang Z. (2013). pH-Sensitive Docetaxel-Loaded d-α-Tocopheryl Polyethylene Glycol Succinate–Poly(β-amino ester) Copolymer Nanoparticles for Overcoming Multidrug Resistance. Biomacromolecules.

[145] Wang D., Tang J., Wang Y., Ramishetti S., Fu Q., Racette K., Liu F. (2013). Multifunctional Nanoparticles Based on a Single-Molecule Modification for the Treatment of Drug-Resistant Cancer. Mol. Pharm..

[146] Leszczyniecka M., Roberts T., Dent P., Grant S., Fisher P.B. (2001). Differentiation therapy of human cancer: Basic science and clinical applications. Pharmacol. Ther..

[147] Lobo N.A., Shimono Y., Qian D., Clarke M.F. (2007). The Biology of Cancer Stem Cells. Annu. Rev. Cell Dev. Biol..

[148] Huang J., Wang K., Wu J., Wang J. (2013). Cancer stem cell theory: Therapeutic implications for nanomedicine. Int. J. Nanomed..

[149] Zhang Y., Zhang H., Wang X., Wang J., Zhang X., Zhang Q. (2012). The eradication of breast cancer and cancer stem cells using octreotide modified paclitaxel active targeting micelles and salinomycin passive targeting micelles. Biomaterials.

[150] Muntimadugu E., Kumar R., Saladi S., Rafeeqi T.A., Khan W. (2016). CD44 targeted chemotherapy for co-eradication of breast cancer stem cells and cancer cells using polymeric nanoparticles of salinomycin and paclitaxel. Colloids Surfaces B Biointerfaces.

[151] Yang Z., Sun N., Cheng R., Zhao C., Liu Z., Li X., Liu J., Tian Z. (2017). pH multistage responsive micellar system with charge-switch and PEG layer detachment for co-delivery of paclitaxel and curcumin to synergistically eliminate breast cancer stem cells. Biomaterials.

[152] Gewirtz D.A. (1999). A critical evaluation of the mechanisms of action proposed for the antitumor effects of the anthracycline antibiotics adriamycin and daunorubicin. Biochem. Pharmacol..

[153] Jung K., Reszka R. (2001). Mitochondria as subcellular targets for clinically useful anthracyclines. Adv. Drug Deliv. Rev..

[154] Mizutani H., Tada-Oikawa S., Hiraku Y., Kojima M., Kawanishi S. (2005). Mechanism of apoptosis induced by doxorubicin through the generation of hydrogen peroxide. Life Sci..

[155] Wu H., Jin H., Wang C., Zhang Z., Ruan H., Sun L., Yang C., Li Y., Qin W., Wang C. (2017). Synergistic Cisplatin/Doxorubicin Combination Chemotherapy for Multidrug-Resistant Cancer via Polymeric Nanogels Targeting Delivery. ACS Appl. Mater. Interfaces.

[156] Emilienne Soma C., Dubernet C., Bentolila D., Benita S., Couvreur P. (2000). Reversion of multidrug resistance by co-encapsulation of doxorubicin and cyclosporin A in polyalkylcyanoacrylate nanoparticles. Biomaterials.

[157] Misra R., Sahoo S.K. (2011). Coformulation of doxorubicin and curcumin in poly(D,L-lactide-co-glycolide) nanoparticles suppresses the development of multidrug resistance in K562 cells. Mol. Pharm..

[158] Zhang J., Li J., Shi Z., Yang Y., Xie X., Lee S.M., Wang Y., Leong K.W., Chen M. (2017). pH-sensitive polymeric nanoparticles for co-delivery of doxorubicin and curcumin to treat cancer via enhanced pro-apoptotic and anti-angiogenic activities. Acta Biomater..

[159] Ma W., Guo Q., Li Y., Wang X., Wang J., Tu P. (2017). Co-assembly of doxorubicin and curcumin targeted micelles for synergistic delivery and improving anti-tumor efficacy. Eur. J. Pharm. Biopharm..

[160] Guo S., Lv L., Shen Y., Hu Z., He Q., Chen X. (2016). A nanoparticulate pre-chemosensitizer for efficacious chemotherapy of multidrug resistant breast cancer. Sci. Rep..

[161] Yuan J.-D., ZhuGe D.-L., Tong M.-Q., Lin M.-T., Xu X.-F., Tang X., Zhao Y.-Z., Xu H.-L. (2018). pH-sensitive polymeric nanoparticles of mPEG-PLGA-PGlu with hybrid core for simultaneous encapsulation of curcumin and doxorubicin to kill the heterogeneous tumour cells in breast cancer. Artif Cells Nanomed. Biotechnol..

[162] Lammers T., Subr V., Ulbrich K., Peschke P., Huber P.E., Hennink W.E., Storm G. (2009). Simultaneous delivery of doxorubicin and gemcitabine to tumors in vivo using prototypic polymeric drug carriers. Biomaterials.

[163] Tai W., Mo R., Lu Y., Jiang T., Gu Z. (2014). Folding graft copolymer with pendant drug segments for co-delivery of anticancer drugs. Biomaterials.

[164] Duan X., Xiao J., Yin Q., Zhang Z., Yu H., Mao S., Li Y. (2013). Smart pH-Sensitive and Temporal-Controlled Polymeric Micelles for Effective Combination Therapy of Doxorubicin and Disulfiram. ACS Nano..

[165] Bae Y., Diezi T.A., Zhao A., Kwon G.S. (2007). Mixed polymeric micelles for combination cancer chemotherapy through the concurrent delivery of multiple chemotherapeutic agents. J. Control Release.

[166] Liao L., Liu J., Dreaden E.C., Morton S.W., Shopsowitz K.E., Hammond P.T., Johnson J.A. (2014). A Convergent Synthetic Platform for Single-Nanoparticle Combination Cancer Therapy: Ratiometric Loading and Controlled Release of Cisplatin, Doxorubicin, and Camptothecin. J. Am. Chem. Soc..

[167] Sun R., Liu Y., Li S.-Y., Shen S., Du X.-J., Xu C.-F., Cao Z.-T., Bao Y., Zhu Y.-H., Li Y.-P. (2015). Co-delivery of all-trans-retinoic acid and doxorubicin for cancer therapy with synergistic inhibition of cancer stem cells. Biomaterials.

[168] Kim H.-O., Kim E., An Y., Choi J., Jang E., Choi E.B., Kukreja A., Kim M.-H., Kang B., Kim D.-J. (2013). A Biodegradable Polymersome Containing Bcl-xL siRNA and Doxorubicin as a Dual Delivery Vehicle for a Synergistic Anticancer Effect. Macromol. Biosci..

[169] Wang Y., Gao S., Ye W.-H., Yoon H.S., Yang Y.-Y. (2006). Co-delivery of drugs and DNA from cationic core–shell nanoparticles self-assembled from a biodegradable copolymer. Nat. Mater..

[170] Yu H., Xu Z., Chen X., Xu L., Yin Q., Zhang Z., Li Y. (2014). Reversal of Lung Cancer Multidrug Resistance by pH-Responsive Micelleplexes Mediating Co-Delivery of siRNA and Paclitaxel. Macromol. Biosci..

[171] Yin Q., Shen J., Zhang Z., Yu H., Chen L., Gu W., Li Y. (2013). Multifunctional Nanoparticles Improve Therapeutic Effect for Breast Cancer by Simultaneously Antagonizing Multiple Mechanisms of Multidrug Resistance. Biomacromolecules.

[172] Patil Y.B., Swaminathan S.K., Sadhukha T., Ma L., Panyam J. (2010). The use of nanoparticle-mediated targeted gene silencing and drug delivery to overcome tumor drug resistance. Biomaterials.

[173] Xiong X.-B., Lavasanifar A. (2011). Traceable Multifunctional Micellar Nanocarriers for Cancer-Targeted Co-delivery of MDR-1 siRNA and Doxorubicin. ACS Nano..

[174] Pan J., Mendes L.P., Yao M., Filipczak N., Garai S., Thakur G.A., Sarisozen C., Torchilin V.P. (2019). Polyamidoamine dendrimers-based nanomedicine for combination therapy with siRNA and chemotherapeutics to overcome multidrug resistance. Eur. J. Pharm. Biopharm..

[175] Prasad V.V., Gopalan R.O. (2015). Continued use of MDA-MB-435, a melanoma cell line, as a model for human breast cancer, even in year, 2014. NPJ Breast Cancer.

[176] Xu X., Xie K., Zhang X.-Q., Pridgen E.M., Park G.Y., Cui D.S., Shi J., Wu J., Kantoff P.W., Lippard S.J. (2013). Enhancing tumor cell response to chemotherapy through nanoparticle-mediated codelivery of siRNA and cisplatin prodrug. Proc. Natl. Acad. Sci. USA.

[177] Kaneshiro T.L., Lu Z.-R. (2009). Targeted intracellular codelivery of chemotherapeutics and nucleic acid with a well-defined dendrimer-based nanoglobular carrier. Biomaterials.

[178] Tekade R.K., Dutta T., Gajbhiye V., Jain N.K. (2009). Exploring dendrimer towards dual drug delivery: pH responsive simultaneous drug-release kinetics. J. Microencapsul..

[179] Miao L., Guo S., Zhang J., Kim W.Y., Huang L. (2014). Nanoparticles with Precise Ratiometric Co-Loading and Co-Delivery of Gemcitabine Monophosphate and Cisplatin for Treatment of Bladder Cancer. Adv. Funct. Mater..

[180] Handali S., Moghimipour E., Rezaei M., Saremy S., Dorkoosh F.A. (2019). Co-delivery of 5-fluorouracil and oxaliplatin in novel poly(3-hydroxybutyrate-co-3-hydroxyvalerate acid)/poly(lactic-co-glycolic acid) nanoparticles for colon cancer therapy. Int. J. Biol. Macromol..

[181] Ni W., Li Z., Liu Z., Ji Y., Wu L., Sun S., Jian X., Gao X. (2019). Dual-Targeting Nanoparticles: Codelivery of Curcumin and 5-Fluorouracil for Synergistic Treatment of Hepatocarcinoma. J. Pharm. Sci..

[182] Song X.R., Cai Z., Zheng Y., He G., Cui F.Y., Gong D.Q., Hou S.X., Xiong S.J., Lei X.J., Wei Y.Q. (2009). Reversion of multidrug resistance by co-encapsulation of vincristine and verapamil in PLGA nanoparticles. Eur. J. Pharm. Sci..

[183] Jain A.K., Thanki K., Jain S. (2013). Co-encapsulation of Tamoxifen and Quercetin in Polymeric Nanoparticles: Implications on Oral Bioavailability, Antitumor Efficacy, and Drug-Induced Toxicity. Mol. Pharm..

[184] Saneja A., Kumar R., Mintoo M.J., Dubey R.D., Sangwan P.L., Mondhe D.M., Panda A.K., Gupta P.N. (2019). Gemcitabine and betulinic acid co-encapsulated PLGA−PEG polymer nanoparticles for improved efficacy of cancer chemotherapy. Mater. Sci. Eng. C.

[185] Wang K., Wen H.-F., Yu D.-G., Yang Y., Zhang D.-F. (2018). Electrosprayed hydrophilic nanocomposites coated with shellac for colon-specific delayed drug delivery. Mater. Des..

[186] Sanchez-Vazquez B., Amaral A.J.R., Yu D.-G., Pasparakis G., Williams G.R. (2017). Electrosprayed Janus Particles for Combined Photo-Chemotherapy. AAPS PharmSciTech..

[187] Zhang L., Zhang M., Zhou L., Han Q., Chen X., Li S., Li L., Su Z., Wang C. (2018). Dual drug delivery and sequential release by amphiphilic Janus nanoparticles for liver cancer theranostics. Biomaterials.

[188] Zhang Y., Huang K., Lin J., Huang P. (2019). Janus nanoparticles in cancer diagnosis, therapy and theranostics. Biomater. Sci..

[189] Hanahan D., Weinberg R.A. (2000). The hallmarks of cancer. Cell.

[190] Malumbres M., Carnero A. (2003). Cell cycle deregulation: a common motif in cancer. Prog. Cell Cycle Res..

[191] Hsu J.L., Hung M.-C. (2016). The role of HER2, EGFR, and other receptor tyrosine kinases in breast cancer. Cancer Metastasis Rev..

[192] Normanno N., De Luca A., Bianco C., Strizzi L., Mancino M., Maiello M.R., Carotenuto A., De Feo G., Caponigro F., Salomon D.S. (2006). Epidermal growth factor receptor (EGFR) signaling in cancer. Gene.

[193] KC R.B., Chandrashekaran V., Cheng B., Chen H., Peña M.M.O., Zhang J., Montgomery J., Xu P. (2014). Redox Potential Ultrasensitive Nanoparticle for the Targeted Delivery of Camptothecin to HER2-Positive Cancer Cells. Mol. Pharm..

[194] Acharya S., Dilnawaz F., Sahoo S.K. (2009). Targeted epidermal growth factor receptor nanoparticle bioconjugates for breast cancer therapy. Biomaterials.

[195] Jin H., Pi J., Zhao Y., Jiang J., Li T., Zeng X., Yang P., Evans C.E., Cai J. (2017). EGFR-targeting PLGA-PEG nanoparticles as a curcumin delivery system for breast cancer therapy. Nanoscale.

[196] Patel J., Amrutiya J., Bhatt P., Javia A., Jain M., Misra A. (2018). Targeted delivery of monoclonal antibody conjugated docetaxel loaded PLGA nanoparticles into EGFR overexpressed lung tumour cells. J. Microencapsul..

[197] Cui X., Sun Y., Shen M., Song K., Yin X., Di W., Duan Y. (2018). Enhanced Chemotherapeutic Efficacy of Paclitaxel Nanoparticles Co-delivered with MicroRNA-7 by Inhibiting Paclitaxel-Induced EGFR/ERK pathway Activation for Ovarian Cancer Therapy. ACS Appl. Mater. Interfaces.

[198] Dhillon A.S., Hagan S., Rath O., Kolch W. (2007). MAP kinase signalling pathways in cancer. Oncogene.

[199] Santarpia L., Lippman S.M., El-Naggar A.K. (2012). Targeting the MAPK–RAS–RAF signaling pathway in cancer therapy. Expert Opin. Ther. Targets.

[200] Basu S., Harfouche R., Soni S., Chimote G., Mashelkar R.A., Sengupta S. (2009). Nanoparticle-mediated targeting of MAPK signaling predisposes tumor to chemotherapy. Proc. Natl. Acad. Sci. USA.

[201] Chen Y., Liu Y.-C., Sung Y.-C., Ramjiawan R.R., Lin T.-T., Chang C.-C., Jeng K.-S., Chang C.-F., Liu C.-H., Gao D.-Y. (2017). Overcoming sorafenib evasion in hepatocellular carcinoma using CXCR4-targeted nanoparticles to co-deliver MEK-inhibitors. Sci. Rep..

[202] Babos G., Biró E., Meiczinger M., Feczkó T., Babos G., Biró E., Meiczinger M., Feczkó T. (2018). Dual Drug Delivery of Sorafenib and Doxorubicin from PLGA and PEG-PLGA Polymeric Nanoparticles. Polymers.

[203] Vivanco I., Sawyers C.L. (2002). The phosphatidylinositol 3-Kinase–AKT pathway in human cancer. Nat. Rev. Cancer.

[204] Harfouche R., Basu S., Soni S., Hentschel D.M., Mashelkar R.A., Sengupta S. (2009). Nanoparticle-mediated targeting of phosphatidylinositol-3-kinase signaling inhibits angiogenesis. Angiogenesis.

[205] Lu X.-Y., Ciraolo E., Stefenia R., Chen G.-Q., Zhang Y., Hirsch E. (2011). Sustained release of PI3K inhibitor from PHA nanoparticles and in vitro growth inhibition of cancer cell lines. Appl. Microbiol. Biotechnol..

[206] Gholizadeh S., Kamps J.A.A.M., Hennink W.E., Kok R.J. (2018). PLGA-PEG nanoparticles for targeted delivery of the mTOR/PI3kinase inhibitor dactolisib to inflamed endothelium. Int. J. Pharm..

[207] Yu H., Pardoll D., Jove R. (2009). STATs in cancer inflammation and immunity: A leading role for STAT3. Nat. Rev. Cancer.

[208] Yu H., Lee H., Herrmann A., Buettner R., Jove R. (2014). Revisiting STAT3 signalling in cancer: New and unexpected biological functions. Nat. Rev. Cancer.

[209] Darnell J.E. (2005). Validating Stat3 in cancer therapy. Nat. Med..

[210] Glienke W., Maute L., Wicht J., Bergmann L. (2009). Curcumin Inhibits Constitutive STAT3 Phosphorylation in Human Pancreatic Cancer Cell lines and Downregulation of Survivin/BIRC5 Gene Expression. Cancer Invest..

[211] Alexandrow M.G., Song L.J., Altiok S., Gray J., Haura E.B., Kumar N.B. (2012). Curcumin: A novel Stat3 pathway inhibitor for chemoprevention of lung cancer. Eur. J. Cancer Prev..

[212] Lim K.J., Bisht S., Bar E.E., Maitra A., Eberhart C.G. (2011). A polymeric nanoparticle formulation of curcumin inhibits growth, clonogenicity and stem-like fraction in malignant brain tumors. Cancer Biol. Ther..

[213] Yallapu M.M., Khan S., Maher D.M., Ebeling M.C., Sundram V., Chauhan N., Ganju A., Balakrishna S., Gupta B.K., Zafar N. (2014). Anti-cancer activity of curcumin loaded nanoparticles in prostate cancer. Biomaterials.

[214] Yallapu M.M., Maher D.M., Sundram V., Bell M.C., Jaggi M., Chauhan S.C. (2010). Curcumin induces chemo/radio-sensitization in ovarian cancer cells and curcumin nanoparticles inhibit ovarian cancer cell growth. J. Ovarian Res..

[215] Molavi O., Mahmud A., Hamdy S., Hung R.W., Lai R., Samuel J., Lavasanifar A. (2010). Development of a Poly(D,L-lactic-co-glycolic acid) Nanoparticle Formulation of STAT3 Inhibitor JSI-124: Implication for Cancer Immunotherapy. Mol. Pharm..

[216] Huang Y.-H., Vakili M., Molavi O., Morrissey Y., Wu C., Paiva I., Soleimani A., Sanaee F., Lavasanifar A., Lai R. (2019). Decoration of Anti-CD38 on Nanoparticles Carrying a STAT3 Inhibitor Can Improve the Therapeutic Efficacy Against Myeloma. Cancers.

[217] Alshamsan A., Haddadi A., Hamdy S., Samuel J., El-Kadi A.O.S., Uludağ H., Lavasanifar A. (2010). STAT3 Silencing in Dendritic Cells by siRNA Polyplexes Encapsulated in PLGA Nanoparticles for the Modulation of Anticancer Immune Response. Mol. Pharm..

[218] Su W.-P., Cheng F.-Y., Shieh D.-B., Yeh C.-S., Su W.-C. (2012). PLGA nanoparticles codeliver paclitaxel and Stat3 siRNA to overcome cellular resistance in lung cancer cells. Int. J. Nanomed..

[219] Das J., Das S., Paul A., Samadder A., Bhattacharyya S.S., Khuda-Bukhsh A.R. (2014). Assessment of drug delivery and anticancer potentials of nanoparticles-loaded siRNA targeting STAT3 in lung cancer, in vitro and in vivo. Toxicol. Lett..

[220] Heo M.B., Lim Y.T. (2014). Programmed nanoparticles for combined immunomodulation, antigen presentation and tracking of immunotherapeutic cells. Biomaterials.

[221] Beh C.W., Seow W.Y., Wang Y., Zhang Y., Ong Z.Y., Ee P.L.R., Yang Y.-Y. (2009). Efficient Delivery of Bcl-2-Targeted siRNA Using Cationic Polymer Nanoparticles: Downregulating mRNA Expression Level and Sensitizing Cancer Cells to Anticancer Drug. Biomacromolecules.

[222] Bertin P.A., Gibbs J.M., Shen C.K.-F., Thaxton C.S., Russin W.A., Mirkin C.A., Nguyen S.T. (2006). Multifunctional Polymeric Nanoparticles from Diverse Bioactive Agents. J. Am. Chem. Soc..

[223] Shen M., Gong F., Pang P., Zhu K., Meng X., Wu C., Wang J., Shan H., Shuai X. (2012). An MRI-visible non-viral vector for targeted Bcl-2 siRNA delivery to neuroblastoma. Int. J. Nanomed..

[224] Sun W., Chen X., Xie C., Wang Y., Lin L., Zhu K., Shuai X. (2018). Co-Delivery of Doxorubicin and Anti-BCL-2 siRNA by pH-Responsive Polymeric Vector to Overcome Drug Resistance in In Vitro and In Vivo HepG2 Hepatoma Model. Biomacromolecules.

[225] Kumar M., Gupta D., Singh G., Sharma S., Bhat M., Prashant C.K., Dinda A.K., Kharbanda S., Kufe D., Singh H. (2014). Novel Polymeric Nanoparticles for Intracellular Delivery of Peptide Cargos: Antitumor Efficacy of the BCL-2 Conversion Peptide NuBCP-9. Cancer Res..

[226] Wang X., Liow S.S., Wu Q., Li C., Owh C., Li Z., Loh X.J., Wu Y.-L. (2017). Codelivery for Paclitaxel and Bcl-2 Conversion Gene by PHB-PDMAEMA Amphiphilic Cationic Copolymer for Effective Drug Resistant Cancer Therapy. Macromol. Biosci..

[227] Kim E., Jung Y., Choi H., Yang J., Suh J.-S., Huh Y.-M., Kim K., Haam S. (2010). Prostate cancer cell death produced by the co-delivery of Bcl-xL shRNA and doxorubicin using an aptamer-conjugated polyplex. Biomaterials.

[228] Ebrahimian M., Taghavi S., Mokhtarzadeh A., Ramezani M., Hashemi M. (2017). Co-delivery of Doxorubicin Encapsulated PLGA Nanoparticles and Bcl-xL shRNA Using Alkyl-Modified PEI into Breast Cancer Cells. Appl. Biochem. Biotechnol..

[229] Ayatollahi S., Salmasi Z., Hashemi M., Askarian S., Oskuee R.K., Abnous K., Ramezani M. (2017). Aptamer-targeted delivery of Bcl-xL shRNA using alkyl modified PAMAM dendrimers into lung cancer cells. Int. J. Biochem. Cell Biol..

[230] Shen J., Yin Q., Chen L., Zhang Z., Li Y. (2012). Co-delivery of paclitaxel and survivin shRNA by pluronic P85-PEI/TPGS complex nanoparticles to overcome drug resistance in lung cancer. Biomaterials.

[231] Jin M., Jin G., Kang L., Chen L., Gao Z., Huang W. (2018). Smart polymeric nanoparticles with pH-responsive and PEG-detachable properties for co-delivering paclitaxel and survivin siRNA to enhance antitumor outcomes. Int. J. Nanomed..

[232] Wang S., Zhang J., Wang Y., Chen M. (2016). Hyaluronic acid-coated PEI-PLGA nanoparticles mediated co-delivery of doxorubicin and miR-542-3p for triple negative breast cancer therapy. Nanomed. Nanotechnol. Biol. Med..

[233] Xu C., Tian H., Wang P., Wang Y., Chen X. (2016). The suppression of metastatic lung cancer by pulmonary administration of polymer nanoparticles for co-delivery of doxorubicin and Survivin siRNA. Biomater. Sci..

[234] Chen W., Zhang M., Shen W., Du B., Yang J., Zhang Q., Chen W., Zhang M., Shen W., Du B. (2019). A Polycationic Brush Mediated Co-Delivery of Doxorubicin and Gene for Combination Therapy. Polymers.

[235] Davoodi P., Srinivasan M.P., Wang C.-H. (2016). Synthesis of intracellular reduction-sensitive amphiphilic polyethyleneimine and poly(ε-caprolactone) graft copolymer for on-demand release of doxorubicin and p53 plasmid DNA. Acta Biomater..

[236] Fodde R., Brabletz T. (2007). Wnt/β-catenin signaling in cancer stemness and malignant behavior. Curr. Opin. Cell Biol..

[237] Franiak-Pietryga I., Maciejewski H., Ziemba B., Appelhans D., Voit B., Robak T., Jander M., Treliński J., Bryszewska M., Borowiec M. (2017). Blockage of Wnt/β-Catenin Signaling by Nanoparticles Reduces Survival and Proliferation of CLL Cells In Vitro-Preliminary Study. Macromol. Biosci..

[238] Gonnissen A., Isebaert S., Haustermans K. (2015). Targeting the Hedgehog signaling pathway in cancer: Beyond Smoothened. Oncotarget.

[239] Chenna V., Hu C., Pramanik D., Aftab B.T., Karikari C., Campbell N.R., Hong S.-M., Zhao M., Rudek M.A., Khan S.R. (2012). A Polymeric Nanoparticle Encapsulated Small-Molecule Inhibitor of Hedgehog Signaling (NanoHHI) Bypasses Secondary Mutational Resistance to Smoothened Antagonists. Mol. Cancer Ther..

[240] Xu Y., Chenna V., Hu C., Sun H.-X., Khan M., Bai H., Yang X.-R., Zhu Q.-F., Sun Y.-F., Maitra A. (2012). Polymeric Nanoparticle-Encapsulated Hedgehog Pathway Inhibitor HPI-1 (NanoHHI) Inhibits Systemic Metastases in an Orthotopic Model of Human Hepatocellular Carcinoma. Clin. Cancer Res..

[241] Kumar V., Mundra V., Mahato R.I. (2014). Nanomedicines of Hedgehog Inhibitor and PPAR-γ Agonist for Treating Liver Fibrosis. Pharm. Res..

[242] Kizaka-Kondoh S., Inoue M., Harada H., Hiraoka M. (2003). Tumor hypoxia: A target for selective cancer therapy. Cancer Sci..

[243] Liu X.-Q., Xiong M.-H., Shu X.-T., Tang R.-Z., Wang J. (2012). Therapeutic Delivery of siRNA Silencing HIF-1 Alpha with Micellar Nanoparticles Inhibits Hypoxic Tumor Growth. Mol. Pharm..

[244] Zhao X., Li F., Li Y., Wang H., Ren H., Chen J., Nie G., Hao J. (2015). Co-delivery of HIF1α siRNA and gemcitabine via biocompatible lipid-polymer hybrid nanoparticles for effective treatment of pancreatic cancer. Biomaterials.

[245] Zhang C., Wang Y.-S., Wu H., Zhang Z.-X., Cai Y., Hou H.-Y., Zhao W., Yang X.-M., Ma J.-X. (2010). Inhibitory efficacy of hypoxia-inducible factor 1α short hairpin RNA plasmid DNA-loaded poly (D, L-lactide-co-glycolide) nanoparticles on choroidal neovascularization in a laser-induced rat model. Gene Ther..

[246] Dang C.V. (2012). MYC on the Path to Cancer. Cell.

[247] Tangudu N.K., Verma V.K., Clemons T.D., Beevi S.S., Hay T., Mahidhara G., Raja M., Nair R.A., Alexander L.E., Patel A.B. (2015). RNA Interference Using c-Myc-Conjugated Nanoparticles Suppresses Breast and Colorectal Cancer Models. Mol. Cancer Ther..

[248] Gabizon A., Shmeeda H., Grenader T. (2012). Pharmacological basis of pegylated liposomal doxorubicin: Impact on cancer therapy. Eur. J. Pharm. Sci..

[249] Albano J.M., Ribeiro LN de M., Couto V.M., Barbosa Messias M., Rodrigues da Silva G.H., Breitkreitz M.C., de Paula E., Pickholz M. (2019). Rational design of polymer-lipid nanoparticles for docetaxel delivery. Colloids Surfaces B Biointerfaces.

[250] Hai T., Wan X., Yu D.-G., Wang K., Yang Y., Liu Z.-P. (2019). Electrospun lipid-coated medicated nanocomposites for an improved drug sustained-release profile. Mater. Des..

[251] Wakaskar R.R. (2018). General overview of lipid–polymer hybrid nanoparticles, dendrimers, micelles, liposomes, spongosomes and cubosomes. J. Drug Target..

[252] Wu X.Y. (2016). Strategies for optimizing polymer-lipid hybrid nanoparticle-mediated drug delivery. Expert Opin. Drug Deliv..

[253] Kaelin W.G. (2005). The Concept of Synthetic Lethality in the Context of Anticancer Therapy. Nat. Rev. Cancer.

[254] Iglehart J.D., Silver D.P. (2009). Synthetic Lethality—A New Direction in Cancer-Drug Development. N. Engl. J. Med..

